# Genetic and oncogenic features of RASGRF fusions

**DOI:** 10.1038/s41698-025-01017-1

**Published:** 2025-07-05

**Authors:** Sreya Das, Daniel S. Lenchner, Ellen Jaeger, Lisa Hunihan, Dana F. DeSantis, Stamatina Fragkogianni, Karyn Ronski, Frederick H. Wilson

**Affiliations:** 1 Department of Internal Medicine, Section of Medical Oncology, Yale School of Medicine, New Haven, CT USA; 2 Department of Genetics, Yale School of Medicine, New Haven, CT USA; 3 Center of Molecular and Cellular Oncology, Yale Cancer Center, Yale School of Medicine, New Haven, CT USA; 4 Tempus AI, Inc, Chicago, IL USA

**Keywords:** Cancer, Genetics, Medical research, Oncology

## Abstract

The identification of recurrent oncogenic drivers has enabled targeted therapeutic strategies for subsets of non-small cell lung carcinoma (NSCLC) and other malignancies. Oncogenic fusions involving the RAS-activating guanine exchange factor (GEF) RASGRF1 are reported in multiple tumors, but their prevalence and genetic heterogeneity remain undefined. Here, we query RNA-seq data from a real-world database of diverse human malignancies and identify 40 tumors with rearrangements involving *RASGRF1* or the related *RASGRF2* predicted to generate chimeric proteins. Half of these fusions occur in NSCLC, pancreatic cancer, and melanoma and are enriched in tumors without other established driver alterations. A subset of RASGRF fusions contains transmembrane partners, and membrane localization enhances RAS activation and transforming activity. Loss of N-terminal PH1 and DH domains in RASGRF fusions also promotes transformation. Although some fusions lack the PH1 but not the DH domain, our functional assays indicate that loss of the PH1 domain alone is insufficient to drive cellular transformation. Our findings provide insights about the tissue distribution, structural diversity, and oncogenic mechanisms of RASGRF fusions. As cell models driven by these fusions are sensitive to MAPK pathway inhibition, oncogenic RASGRF fusions may represent a therapeutic target in rare molecular subsets of cancer.

## Introduction

Dysregulated RAS signaling is a hallmark of tumorigenesis in multiple solid tumor malignancies^1^. Multiple genetic mechanisms of aberrant RAS signaling have been described, including activating mutations in RAS isoforms (KRAS, HRAS, and NRAS) and activating alterations in receptor tyrosine kinases (RTKs) that promote RAS signaling (including EGFR, MET and TRK)^2–4^. RAS GTPase-activating proteins (GAPs) stimulate GTP hydrolysis to reduce levels of active GTP-bound RAS, while RAS guanine exchange factors (GEFs) promote RAS activation by facilitating dissociation of GDP from RAS^5^. Loss-of-function alterations in the RAS-GAPs NF1 and RASA1 that promote RAS activation have been reported in non-small cell lung carcinoma (NSCLC)^6^.

Chromosomal rearrangements can precipitate chimeric fusion proteins that promote cellular transformation and oncogenesis^7,8^. Most of the well-characterized oncogenic fusions with FDA-approved targeted therapies involve RTKs such as ABL, ALK, FLT3, RET, FGFR, and NTRK. However, fusions involving other protein classes have also been described, including RTK ligands (NRG1 and NRG2)^9,10^, the serine-threonine kinase BRAF^11^, and transcriptional activators (such as ERG in prostate cancer^12^, MAML2 in mucoepidermoid carcinoma^13^, and NUT in NUT midline carcinoma^14^). A subset of oncogenic fusions without FDA-approved targeted therapies might still be actionable. For example, repurposing of ALK inhibitors may be a viable therapeutic strategy for advanced NSCLC driven by fusions involving LTK (which is structurally similar to ALK)^15^. Targeting pathways aberrantly activated by a fusion oncoprotein can also be feasible, as demonstrated by clinical activity of HER3-directed therapies in solid tumors with NRG1 fusions (which activate HER3 signaling)^16–18^.

We and others recently reported a distinct class of oncogenic fusions involving the RAS-GEF RASGRF1 in NSCLC, pancreatic ductal adenocarcinoma (PDAC), melanoma, and sarcoma^19–21^. We previously demonstrated that RASGRF1 fusions increase cellular levels of GTP-bound RAS, induce mitogen-activated protein kinase (MAPK) signaling, promote cellular transformation, and drive in vivo tumorigenesis^21^. Cells dependent on RASGRF1 fusions are sensitive to MAPK inhibition. In addition to RASGRF1 fusions, analogous rearrangements involving the related RAS-GEF RASGRF2 have been reported in melanomas and melanocytic neoplasms lacking known driver alterations^22^. Here, we aimed to define the prevalence, tissue distribution, structural diversity, and oncogenic features of RASGRF1 and RASGRF2 fusions.

## Results

### Prevalence of RASGRF fusions in solid tumor malignancies

We previously reported three RASGRF1 fusions: (1) an *OCLN–RASGRF1* fusion in an otherwise driver-negative lung adenocarcinoma from an individual with no smoking history, (2) an *SLC4A4–RASGRF1* fusion in a KRAS wild-type human PDAC cell line, and (3) an *IQGAP1–RASGRF1* fusion from a giant cell sarcoma characterized in The Cancer Genome Atlas (TCGA)^21^. Five additional RASGRF1 fusions have also been reported from NSCLC (*TMEM87A-RASGRF1*)^19^, acute myeloid leukemia (*TMEM154-RASGRF1*)^23^, melanoma (*ABC22-RASGRF1*)^20^, and melanocytic neoplasms with spitzoid features (*CD63-RASGRF1* and *EHBP1-RASGRF1*)^20^. In addition, five RASGRF2 fusions have been identified in melanoma and melanocytic proliferations, including *ATP2B4–RASGRF2* (*n* = 3) and *ERBIN–RASGRF2* (*n* = 2), although no functional characterization of RASGRF2 fusions has been described^22^.

**Fig. 1:**
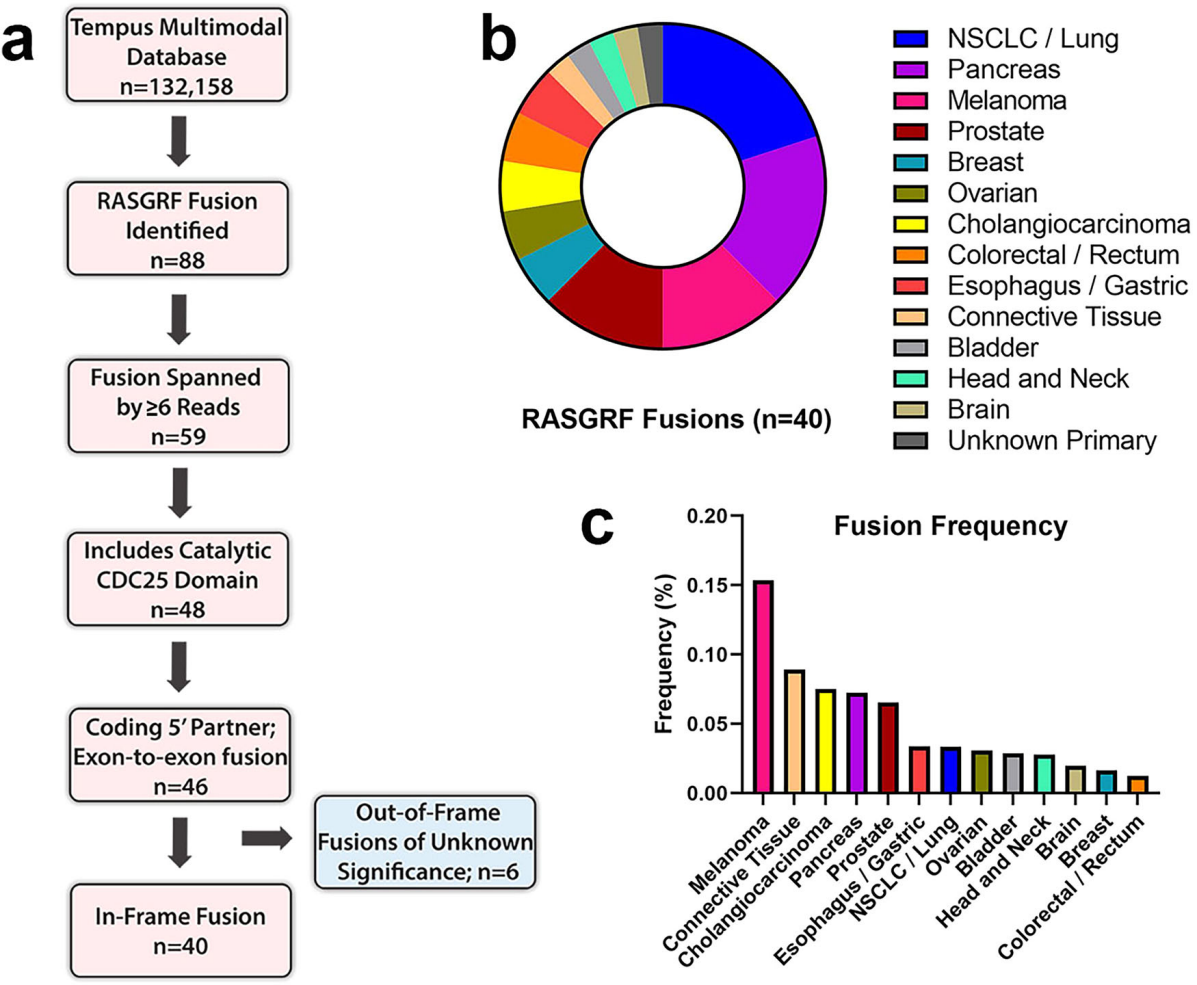
Identification of RASGRF fusions. **a** Schematic overview of the approach to identify RASGRF fusions. **b** Tumor types from which RASGRF fusions were identified. **c** Frequency of RASGRF fusions across tumor types.

To gain insight into the frequency and structural characteristics of RASGRF fusions, we queried 132,158 tumors with RNA-seq data from the Tempus real-world multimodal database. Diverse solid tumor malignancies were represented, with lung, colorectal, breast, pancreatic, and prostate cancers comprising approximately half of the samples (Supplementary Fig. 1). RASGRF fusions were initially detected in 639 RNA samples. These were subjected to rigorous filtering processes to exclude potential technical artifacts, resulting in 88 well-supported RASGRF fusions (Fig. 1a). Tumors with at least 6 sequencing reads spanning the fusion endpoints (*n* = 59) were considered for further evaluation. We next examined these fusions to identify those that preserved the catalytic CDC25 domain responsible for RAS nucleotide exchange. Eleven fusions lacked an intact CDC25 domain, leaving 48 fusions. Two of the 48 fusions were eliminated from further consideration, as one did not appear to involve a coding 5’ gene, and the other fusion included intronic sequence. Of the 46 remaining fusions, 6 were not predicted to preserve the open reading frame of the rearranged genes, and their functional significance is unknown (Supplementary Table 1). The remaining 40 in-frame fusions were considered candidate functional RASGRF fusions (Supplementary Table 2). Half of the 40 fusions were identified in NSCLC, PDAC, and melanoma, with the remainder found in other solid tumor malignancies (Fig. 1b). In this dataset, RASGRF fusions had the highest frequency in melanoma (0.15%) and occurred in <0.1% of other malignancies (Fig. 1c).

### Genomic structure of identified RASGRF1 and RASGRF2 fusions

**Fig. 2:**
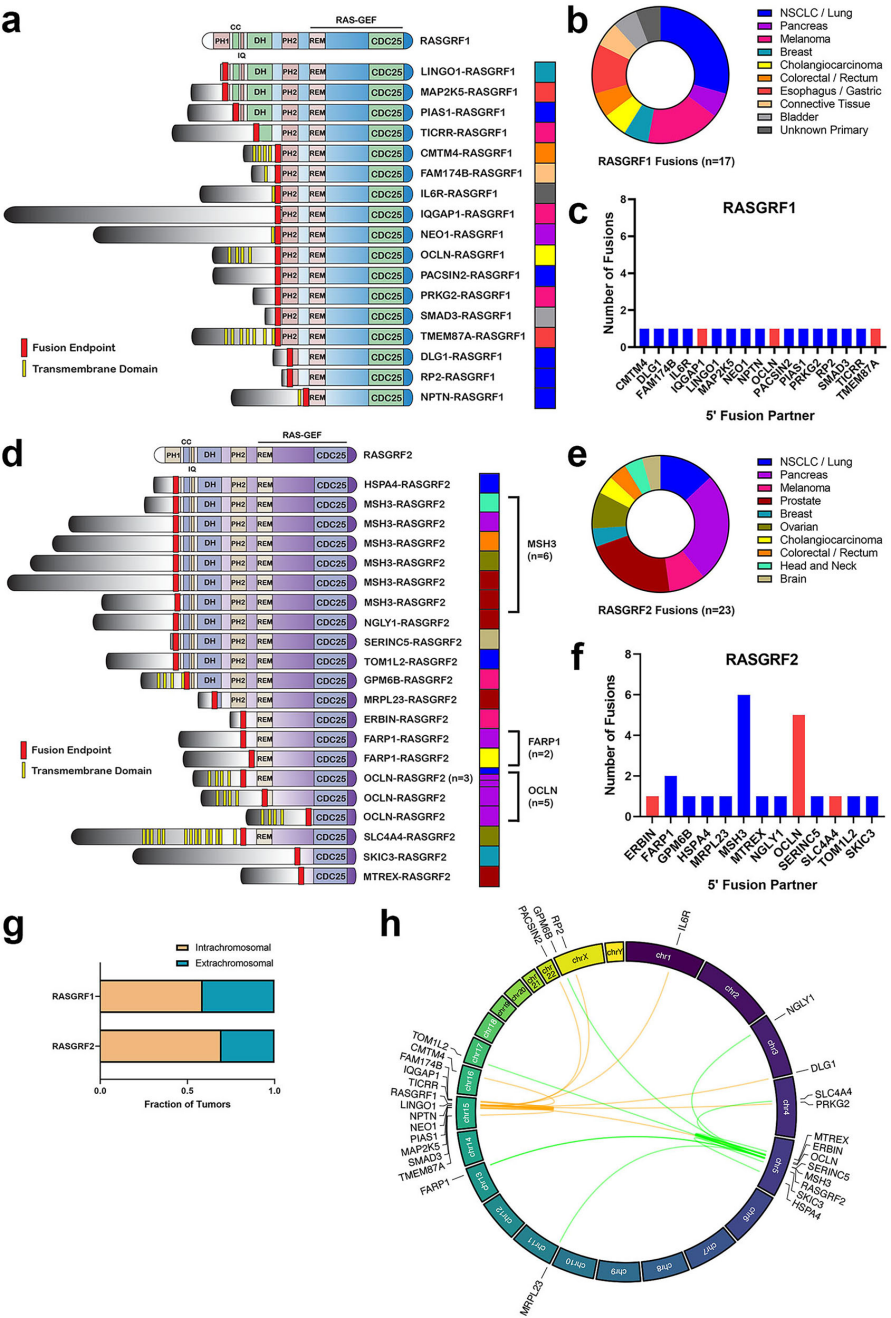
Structural diversity of RASGRF fusions. **a** Functional domains of wild-type RASGRF1 (top) and 17 identified RASGRF1 fusions. Tumor type for each fusion is indicated by the colored bars on the right (using the color scheme in Fig. 1). PH1 pleckstrin homology domain 1, CC coiled coil domain, IQ isoleucine–glutamine domain, DH dbl-homology region, PH2 pleckstrin homology domain 2, REM Ras-exchanger stabilization motif domain. **b** Tumor types from which RASGRF1 fusions were identified. Tumor type for each fusion is indicated by the colored bars on the right. **c** Frequency of 5’ fusion partners among the 17 RASGRF1 fusions. 5’ partners indicated in red have been previously reported in other RASGRF1 fusions. **d** Functional domains of wild-type RASGRF2 (top) and 23 identified RASGRF2 fusions. **e** Tumor types from which RASGRF2 fusions were identified. **f** As in c, except for RASGRF2 fusions. **g** Fraction of intrachromosomal and extrachromosomal RASGRF fusions. **h** Circos plot demonstrating chromosomal location of *RASGRF1*, *RASGRF2*, and identified fusion partners.

We identified 17 RASGRF1 fusions, including 5 in NSCLC, 3 in melanoma, and 1 in PDAC (Fig. 2a, b). Some were identified from tumors in which RASGRF1 fusions have not previously been reported, including cholangiocarcinoma, colorectal, gastroesophageal, and bladder cancers. The 17 fusions each contain a unique 5’ fusion partner, including three (*IQGAP1*, *OCLN*, and *TMEM87A*) previously identified in other RASGRF1 fusions (Fig. 2c). The *TMEM87A-RASGRF1* fusion identified from a gastroesophageal malignancy shares the same fusion endpoints as a previously reported *TMEM87A-RASGRF1* fusion from NSCLC^19^. We previously reported an *OCLN–RASGRF1* fusion from an NSCLC and an *IQGAP1–RASGRF1* fusion from a sarcoma^21^. These differ in their fusion endpoints from the *OCLN–RASGRF1* and *IQGAP1–RASGRF1* fusions reported here from a cholangiocarcinoma and a melanoma, respectively (although the segment of *OCLN* in both *OCLN–RASGRF1* fusions is the same).

We identified 23 RASGRF2 fusions (Fig. 2d). To our knowledge, RASGRF2 fusions have previously been reported only in melanoma and melanocytic proliferations^22^. We identified six RASGRF2 fusions in PDAC, five in prostate cancers, and three in NSCLC/lung cancers (Fig. 2e). Additional RASGRF2 fusions were found in other solid tumor malignancies including melanoma and ovarian cancer. Among the 23 RASGRF2 fusions, we identified 13 distinct 5’ fusion partners (Fig. 2f). Three of these have been previously reported in other RASGRF fusions (*OCLN* and *SLC4A4* in RASGRF1 fusions and *ERBIN* in RASGRF2 fusions)^20,21^.

In total, among the 40 identified fusions, there were 29 unique 5’ fusion partners, including 24 not previously reported. Only 3 of the 29 fusion partners were identified in more than one fusion. These include *FARP1* (found in 2 RASGRF2 fusions from cholangiocarcinoma and PDAC) and *MSH3* (found in 6 RASGRF2 fusions from colorectal, ovarian, head and neck, pancreatic, and prostate cancers; Fig. 2f). In addition, *OCLN* was the fusion partner in a RASGRF1 fusion from cholangiocarcinoma, 5 RASGRF2 fusions from PDAC and NSCLC, and was previously reported in another RASGRF1 fusion from NSCLC by our group^21^. *OCLN* encodes the tight junction transmembrane protein occludin. In all 6 fusions involving *OCLN* (and in the *OCLN–RASGRF1* we previously reported), the fusion endpoint occurs within the C-terminal cytoplasmic tail of occludin. *MSH3* was the most common RASGRF2 fusion partner, identified in 6 fusions. Over half of RASGRF1 and RASGRF2 fusions arose from intrachromosomal gene rearrangements (Fig. 2g, h). The relatively high frequency of RASGRF2 fusions involving *MSH3* may be due to the location of *MSH3* immediately proximal to *RASGRF2* on chromosome 5q (Fig. 2h). Two genes (*OCLN* and *SLC4A4*) have been identified as fusion partners in both RASGRF1 and RASGRF2 fusions (with *SLC4A4–RASGRF1* previously reported by our group in PDAC)^21^.

### Demographic features and co-occurring genetic alterations

**Fig. 3:**
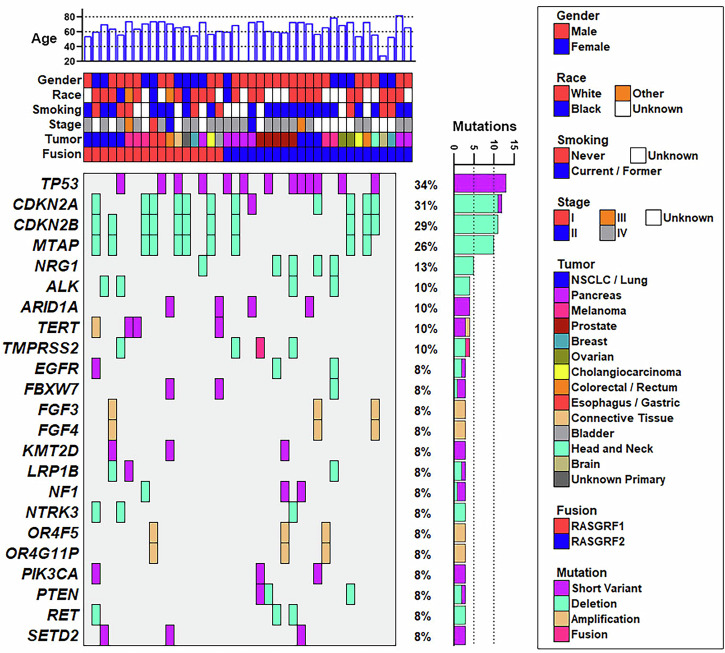
Co-mutation plot of tumors harboring RASGRF fusions. Available demographic and co-mutation data are shown for the 40 tumors identified in this study that harbor in-frame RASGRF fusions. Each column represents a tumor. Genes with co-occurring alterations in at least 3 of the 40 tumors are shown. Co-occurring alterations include somatic pathogenic/likely pathogenic short variant alterations, copy number deletions, copy number amplifications, and fusions. Co-mutation data were not available for 2 of the 40 tumors (shown in the far right), which were subjected to RNA-seq but not DNA sequencing. One pancreatic tumor harboring a RASGRF2 fusion was obtained from a patient of unknown age (corresponding to the sample with no age displayed at the top of the figure).

Among subjects with tumors harboring RASGRF fusions, the median age at diagnosis was 67 years, ranging from age 28–82 (Table 1; Fig. 3). One subject’s age was unknown. Of note, 26 of 40 tumors (65%) with RASGRF fusions occurred in males (Table 1; Fig. 3). Most individuals with tumors harboring RASGRF fusions were Caucasian, although information about race was not available for 14 subjects (Table 1; Fig. 3). Eight subjects were never-smokers, while 22 were current or former smokers. Smoking history was not available for the other 10 subjects. Among individuals with NSCLC/lung cancers harboring RASGRF fusions, 2 were never-smokers while 6 were current or former smokers (Fig. 3; Supplementary Table 3; Supplementary Fig. 2). The *NPTN-RASGRF1* and *RP2-RASGRF1* fusions were identified in NSCLCs from the 2 never-smokers (Supplementary Fig. 2). Most fusions were identified in tumors from individuals with Stage IV malignancy, although staging information was not available for 14 subjects (Table 1; Fig. 3).

**Table 1:** Demographic features of subjects with RASGRF fusions

Characteristic	Subjects (*n* = 40) number (%)
Age at diagnosis	
Median (range) in years	67 (28–82)
Unknown	1
Gender	
Male	26 (65%)
Female	14 (35%)
Race	
White	20 (76.9%)
Black or African-American	4 (15.4%)
Asian	0 (0%)
Pacific Islander	0 (0%)
American Indian	0 (0%)
Other race	2 (7.7%)
Unknown	14
Smoking history	
Never-smoker	8 (26.7%)
Current or Former Smoker	22 (73.3%)
Unknown	10
Stage at sample collection	
I	1 (3.8%)
II	2 (7.7%)
III	2 (7.7%)
IV	21 (80.8%)
Unknown	14

We reviewed histologic subtypes of NSCLC/lung cancers with RASGRF fusions. The data were ascertained from abstracted clinical records (including pathology reports) and were not independently verified. Five of the eight NSCLC/lung cancers harboring a RASGRF fusion were adenocarcinomas (corresponding to the NSCLCs containing *PACSIN2-RASGRF1*, *DLG1-RASGRF1*, *RP2-RASGRF1*, *NPTN-RASGRF1*, and *OCLN–RASGRF2*; Supplementary Fig. 2). The NSCLC with *PIAS1-RASGRF1* was a squamous cell carcinoma, while *HSPA4-RASGRF2* was identified in a large cell neuroendocrine carcinoma of the lung. The histologic subtype of the NSCLC containing *TOM1L2-RASGRF2* is unknown.

We examined co-occurring gene alterations in tumors with RASGRF fusions (Fig. 3). DNA sequencing data were available for 38 of the 40 tumors with RASGRF fusions (data were unavailable from the PDAC harboring *FARP1-RASGRF2* and from one of the PDACs with *OCLN–RASGRF2*). Many of the identified co-occurring alterations are common in cancer. Thirteen tumors with RASGRF fusions had *TP53* alterations. Loss of *CDKN2A*, *CDKN2B*, and/or *MTAP* on chromosome 9p was observed in 12 tumors. *TERT* alterations were found in 4 tumors. Genetic alterations involving other regulators or downstream effectors of RAS signaling were also noted (*NF1*, *PIK3CA*, *PTEN*).

We examined whether RASGRF fusions might be enriched in melanoma, PDAC, and NSCLC/lung cancers lacking established oncogenic drivers in these tumor types. Among 5 melanomas with RASGRF fusions in our dataset, none had co-occurring BRAF or NRAS alterations (which are found in ~50–60% and ~15–25% of melanomas, respectively; Supplementary Fig. 2)^24,25^. Activating KRAS mutations are identified in >90% of PDACs^26^. Among 7 PDACs with a RASGRF fusion, only one (with an *MSH3-RASGRF2* fusion) harbored a co-occurring KRAS mutation (G12D; Supplementary Fig. 2). Eight lung cancers were found to have a RASGRF fusion. One NSCLC with an *NPTN-RASGRF1* fusion also harbored both a classic activating EGFR Exon 19 deletion and an *ADPGK-NTRK3* fusion, both established oncogenic drivers in NSCLC (Supplementary Fig. 2). Of note, this patient had previously been treated with the EGFR inhibitor osimertinib at the time of sample acquisition, raising the possibility that the *NPTN–RASGRF1* and *ADPGK–NTRK3* fusions could represent acquired mechanisms of resistance to osimertinib. Unfortunately, a sample from this patient’s tumor prior to osimertinib administration was not available to determine if the fusions were present at that time. Known oncogenic drivers were not identified in the other seven NSCLC/lung cancers with a RASGRF fusion.

### Plasma membrane localization enhances RAS activation mediated by RASGRF fusions

Seven of 17 RASGRF1 fusions (41%) and 7 of 23 RASGRF2 fusions (30%) identified in this study include transmembrane N-terminal fusion partners with the fusion endpoint predicted to occur within the cytoplasm. Among eight previously reported RASGRF1 fusions, six contain a membrane-spanning N-terminal fusion partner^19–21^. In addition, three of the five previously reported RASGRF2 fusions also involve a transmembrane fusion partner^22^. As membrane association is required for RAS activation, we hypothesized that membrane localization of RASGRF fusions may facilitate RAS activation by bringing the catalytic RAS-GEF domain in proximity with membrane-associated RAS. We tested this by evaluating the effect of (1) disrupting plasma membrane localization of a membrane-spanning RASGRF1 fusion and (2) forcing membrane localization of wild-type RASGRF1 and a RASGRF1 fusion without a transmembrane N-terminal partner.

**Fig. 4:**
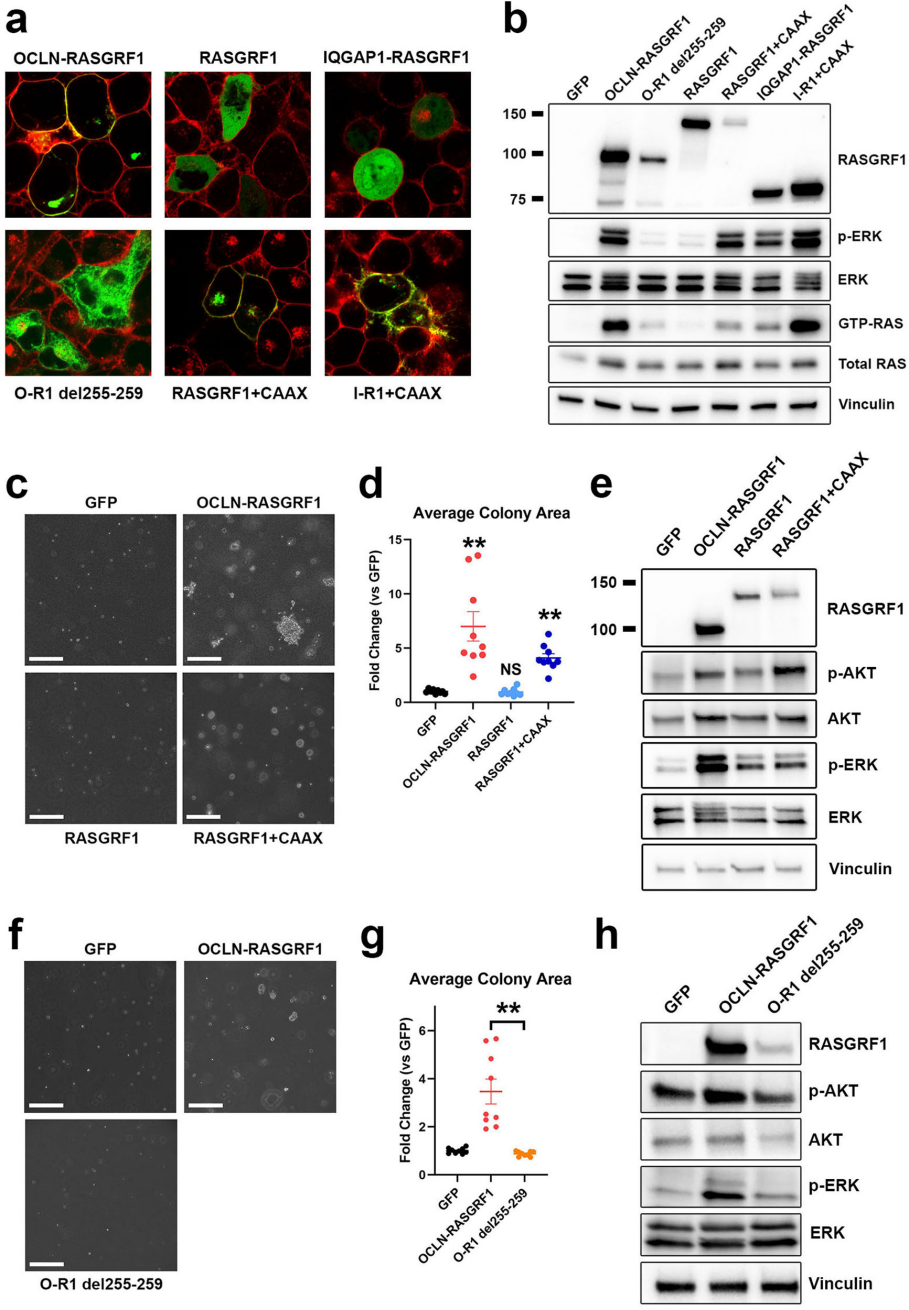
Effect of membrane localization of RASGRF1 fusions on RAS activation. **a** Confocal imaging of live HEK 293 T cells expressing the indicated GFP-tagged cDNAs (green). Prior to imaging, CellMask Plasma Membrane stain was used to label plasma membranes (red). **b** Western immunoblotting of lysates from HEK 293 T cells expressing the indicated cDNAs to assess cellular levels of active RAS (GTP-RAS) and ERK activation (p-ERK). Molecular weight is indicated in kDa on the left. O-R1, OCLN–RASGRF1; I-R1, IQGAP1–RASGRF1. **c** Anchorage-independent proliferation of NIH3T3 cells expressing the indicated cDNAs in soft agar assays. Cells were cultured in soft agar for 19 days. Images are representative of 3 independent experiments. The white marker denotes 300 μm. **d** Quantification of average colony area for NIH3T3 cells expressing the indicated cDNAs (normalized to GFP) after 19–21 days. Mean and standard error for three independent experiments (with three replicates each) are shown. **, *P* < 0.01 by two-tailed t-test compared to GFP. NS not significant (compared to GFP). **e** Western immunoblotting of lysates from NIH3T3 cells expressing the indicated cDNAs. **f** As in **c**, except cells were cultured in soft agar for 15 days. **g** Quantification of average colony area for NIH3T3 cells expressing the indicated cDNAs (normalized to GFP) after 15–19 days. Mean and standard error for three independent experiments (with three replicates each) are shown. **, *P* < 0.01 by two-tailed *t*-test. **h** Western immunoblotting of lysates from NIH3T3 cells expressing the indicated cDNAs.

We previously showed that an OCLN–RASGRF1 fusion (which includes all four transmembrane domains of occludin) localizes to the plasma membrane when ectopically expressed in HEK 293 T cells^21^. Amino acid deletions within the 4th transmembrane domain (TM4) of occludin abolish plasma membrane localization^27^. We used site-directed mutagenesis to delete a 5-amino acid segment of TM4 in OCLN–RASGRF1 (OCLN–RASGRF1 del255–259; see Methods and Supplementary Table 4). We used live cell imaging with confocal microscopy to confirm that this alteration disrupts plasma membrane localization of ectopically expressed, GFP-tagged OCLN–RASGRF1 in HEK 293 T cells (Fig. 4a).

Prior studies demonstrated that forced membrane localization of the isolated catalytic domain of RASGRF1 with a C-terminal membrane targeting sequence (CAAX, where A represents an aliphatic amino acid and X is serine or methionine) promotes transforming activity^28^. We previously reported an oncogenic IQGAP1–RASGRF1 fusion from a sarcoma predicted to include only the first 52 amino acids of IQGAP1 that lacks membrane-spanning domains (and is distinct from the IQGAP1–RASGRF1 fusion identified from a melanoma in the present study)^21^. We introduced GFP-tagged full-length RASGRF1 or the previously reported IQGAP1–RASGRF1 into HEK 293 T cells and assessed subcellular localization with live cell imaging using confocal microscopy. We observed diffuse cytoplasmic expression of both RASGRF1 and IQGAP1–RASGRF1 (Fig. 4a). We next added a C-terminal CAAX peptide to GFP-tagged RASGRF1 and IQGAP1–RASGRF1 and confirmed plasma membrane localization of these constructs in HEK 293 T cells (Fig. 4a).

We next evaluated the capacity of these RASGRF1 constructs to activate RAS signaling in HEK 293 T cells (Fig. 4b). We noted a marked decrease in levels of GTP-RAS and MAPK signaling with disruption of TM4 (OCLN–RASGRF1 del255–259) compared to OCLN–RASGRF1, suggesting that disruption of plasma membrane localization reduces RAS activation. Ectopic expression of full-length RASGRF1 minimally increased levels of GTP-RAS and MAPK signaling compared to cells expressing GFP as a control (Fig. 4b). However, the addition of a C-terminal CAAX sequence to full-length RASGRF1 increased GTP-RAS and markedly increased MAPK activation (Fig. 4b). These findings indicate that forced membrane localization increases RAS activation mediated by full-length RASGRF1. Similar to our findings with wild-type RASGRF1, the addition of a C-terminal CAAX peptide to IQGAP1–RASGRF1 increased RAS activation (Fig. 4b).

We next evaluated the transforming potential of these RASGRF1 constructs. We introduced them into NIH3T3 mouse fibroblasts and assessed anchorage-independent proliferation in soft agar. Ectopic expression of full-length RASGRF1 in NIH3T3 cells did not promote anchorage-independent proliferation (Fig. 4c, d). Consistent with findings in HEK 293 T, RASGRF1 only modestly promoted RAS signaling (as evidenced by ERK and AKT activation) compared to GFP control (Fig. 4e). The addition of the C-terminal CAAX peptide to RASGRF1 promoted anchorage-independent proliferation but to a lesser extent than observed with OCLN–RASGRF1, consistent with their relative effects on RAS activation in HEK 293 T cells (Fig. 4c, d). Conversely, disruption of TM4 of OCLN–RASGRF1 (OCLN–RASGRF1 del255–259) abrogated anchorage-independent proliferation and reduced RAS signaling (Fig. 4f–h). Together, the findings indicate that membrane localization of wild-type RASGRF1 and RASGRF1 fusions enhances RAS activation and cellular transformation.

### OCLN–RASGRF2 fusions promote RAS activation and cellular transformation

**Fig. 5:**
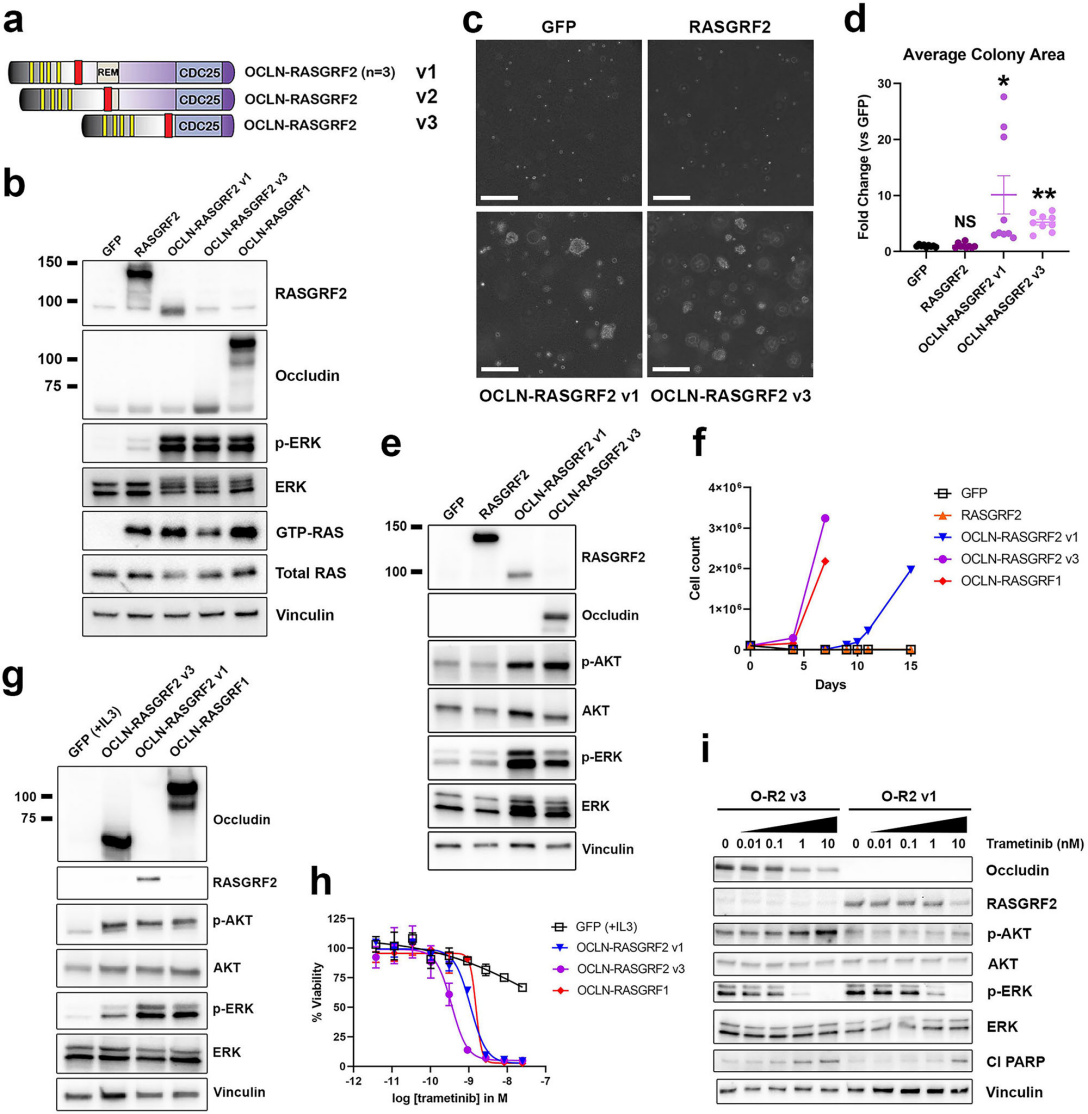
Functional characterization and MEK inhibitor sensitivity of RASGRF2 fusions. **a** Structure of five OCLN–RASGRF2 fusions, as in Fig. 2d. Three distinct fusion variants (v1–v3) were identified. **b** Western immunoblotting of lysates from HEK 293 T cells expressing the indicated cDNAs to assess cellular levels of active RAS (GTP-RAS) and ERK activation (p-ERK). OCLN–RASGRF1 is included as a positive control and comparator for RASGRF2 fusion constructs. OCLN–RASGRF2 v1 contains the epitope recognized by the RASGRF2 antibody but not the occludin antibody. OCLN–RASGRF2 v3 contains the epitope recognized by the occludin antibody but not the RASGRF2 antibody. Molecular weight is indicated in kDa on the left. **c** Anchorage-independent proliferation of NIH3T3 cells expressing the indicated cDNAs in soft agar assays. Cells were cultured in soft agar for 19 days. Images are representative of three independent experiments. The white marker denotes 300 μm. **d** Quantification of average colony area for NIH3T3 cells expressing the indicated cDNAs (normalized to GFP) after 19–21 days. Mean and standard error for three independent experiments (with three replicates each) are shown. These data were collected in parallel with those shown in Fig. 4d using the same GFP control. *, *P* < 0.05. **; *P* < 0.01 by two-tailed *t*-test compared to GFP. NS not significant (compared to GFP). **e** Western immunoblotting of lysates from NIH3T3 cells expressing the indicated cDNAs. **f** Proliferation of Ba/F3 cells expressing the indicated cDNAs after withdrawal of IL3 at Day 0. The experiment was performed 3 times. **g** Western immunoblotting of lysates from Ba/F3 cells expressing the indicated cDNAs. Cells expressing GFP were cultured in the presence of 1 ng/ml IL3. Cells expressing the other cDNAs were cultured in the absence of IL3. **h** Ba/F3 cells expressing the indicated cDNAs were treated with trametinib at the indicated concentrations. After 4 days, cell viability was determined with Cell Titer-Glo. Cells expressing GFP were cultured with 1 ng/mL IL3. Mean and standard error are shown, and the experiment was performed 3 times. **i** Western immunoblotting of lysates from Ba/F3 cells expressing OCLN–RASGRF2 v1 or OCLN–RASGRF2 v3 treated with trametinib at the indicated concentrations for 24 h after serum starvation for 4 h. Cl PARP cleaved PARP.

Three *ATP2B4–RASGRF2* fusions and two *ERBIN–RASGRF2* fusions have been previously reported in melanocytic proliferations (*n* = 4) and a single melanoma lacking other RAS-activating oncogenic alterations^22^. However, no functional characterization of RASGRF2 fusions has been reported. Among the RASGRF2 fusions identified from our dataset, the most common recurrent rearrangement fused Exon 4 of *OCLN* with Exon 13 of *RASGRF2* (*n* = 3; Fig. 2d). We designated this *OCLN–RASGRF2* fusion as variant 1 (v1; Fig. 5a). Among the identified RASGRF2 fusions, another *OCLN–RASGRF2* variant fusing Exon 5 of *OCLN* with Exon 20 of *RASGRF2* (which we designated v3) contains the smallest segment of RASGRF2 (Fig. 5a). The catalytic RAS-GEF domain consists of the CDC25 domain (which catalyzes nucleotide exchange) and the Ras-exchange motif (REM) domain (which binds to RAS substrates)^29–31^. OCLN–RASGRF2 v3 includes the CDC25 domain but lacks the REM domain. As OCLN–RASGRF2 v1 represents the most common RASGRF2 fusion in our dataset and v3 contains the smallest segment of RASGRF2, we generated cDNAs encoding these variants for functional characterization (see Methods).

We ectopically expressed OCLN–RASGRF2 v1, OCLN–RASGRF2 v3, full-length RASGRF2, or GFP in HEK 293 T cells. We confirmed ectopic expression of RASGRF2 and RASGRF2 fusions using antibodies against RASGRF2 and occludin (Fig. 5b). A RASGRF2 antibody was used to confirm expression of RASGRF2 and OCLN–RASGRF2 v1. The epitope recognized by this antibody is absent from OCLN–RASGRF2 v3, and an antibody recognizing occludin was therefore used to confirm expression of this fusion. Of note, the epitope recognized by the occludin antibody is absent from OCLN–RASGRF2 v1; thus, that fusion is not detected with this antibody. We next assessed levels of RAS activation associated with these constructs. Ectopic expression of RASGRF2 promoted increased levels of GTP-RAS and a modest increase in MAPK signaling compared to GFP, while both RASGRF2 fusions promoted a marked increase in RAS activation (comparable to that observed with OCLN–RASGRF1; Fig. 5b).

We next introduced these cDNAs into NIH3T3 cells and assessed anchorage-independent proliferation in soft agar. Like full-length RASGRF1, ectopic expression of full-length RASGRF2 did not promote anchorage-independent proliferation (Fig. 5c, d). In contrast, RASGRF2 fusions promoted robust colony formation relative to GFP or RASGRF2 (Fig. 5c, d). Ectopic expression of RASGRF2 fusions in NIH3T3 cells increased MAPK and PI3K signaling, consistent with RAS activation (Fig. 5e).

To further demonstrate their transforming activity, we expressed both OCLN–RASGRF2 fusions in Ba/F3 cells (a mouse hematopoietic cell line dependent on IL3) and assessed the capacity of the fusions to promote IL3-independent proliferation. Upon withdrawal of IL3 from culture media, Ba/F3 cells expressing OCLN–RASGRF2 fusions (but not GFP or RASGRF2) demonstrated robust proliferation (Fig. 5f). We confirmed ectopic expression of OCLN–RASGRF2 fusions, which upregulated MAPK and PI3K signaling (Fig. 5g). We previously demonstrated that both Ba/F3 cells driven by OCLN–RASGRF1 and a pancreatic ductal adenocarcinoma cell line (PaCaDD137) with an endogenous SLC4A4–RASGRF1 fusion are highly sensitive to targeting of the MAPK pathway with the MEK inhibitor trametinib, while relatively insensitive to PI3K inhibition^21^. We tested the sensitivity of Ba/F3 cells expressing OCLN–RASGRF2 fusions compared to parental Ba/F3 cells (supplemented with IL3) and to OCLN–RASGRF1 (as a positive control). Like OCLN–RASGRF1, Ba/F3 cells driven by OCLN–RASGRF2 v1 or OCLN–RASGRF2 v3 were markedly sensitive to trametinib (Fig. 5h). Parental Ba/F3 cells cultured with IL3 were much less sensitive, suggesting the effect is not due to non-specific toxicity of trametinib in Ba/F3 (Fig. 5h). Trametinib promoted apoptosis in Ba/F3 cells driven by OCLN–RASGRF2 fusions, as evidenced by induction of PARP cleavage with increasing trametinib concentrations (Fig. 5i).

### Genetic features of RASGRF fusions associated with transforming phenotypes

Despite lacking membrane-spanning domains, IQGAP1–RASGRF1 promoted increased RAS activation compared to full-length RASGRF1 (Fig. 4b), suggesting that loss of RASGRF1 N-terminal domains may augment catalytic activity. An autoinhibitory mechanism involving the N-terminal Pleckstrin homology (PH) and Dbl homology (DH) domains of the related RAS-GEF SOS1 has been described^32–35^. RASGRF1 and RASGRF2 contain two PH domains (PH1 and PH2) in addition to a DH domain in the N-terminus (Fig. 2a, d), and there are reports suggesting RASGRFs may have an analogous autoinhibitory mechanism with loss of N-terminal domains promoting RAS signaling^29,36,37^. There are conflicting studies, however, and it has also been reported that the DH domain of RASGRF1 is required for RAS activation^38,39^. RASGRF1 fusions previously shown by us and others to promote cellular transformation lack both the PH1 and DH domains^19,21^. Most of these previously reported fusions also involve membrane-spanning N-terminal fusion partners. The sole exception is the IQGAP1–RASGRF1 fusion we previously reported from a sarcoma, which does not contain membrane-spanning domains but still promotes cell transformation and tumorigenesis in vivo^21^. This suggests that disruption of an autoinhibitory mechanism in RASGRF1 as a result of the fusion may be sufficient to promote cell transformation.

**Fig. 6:**
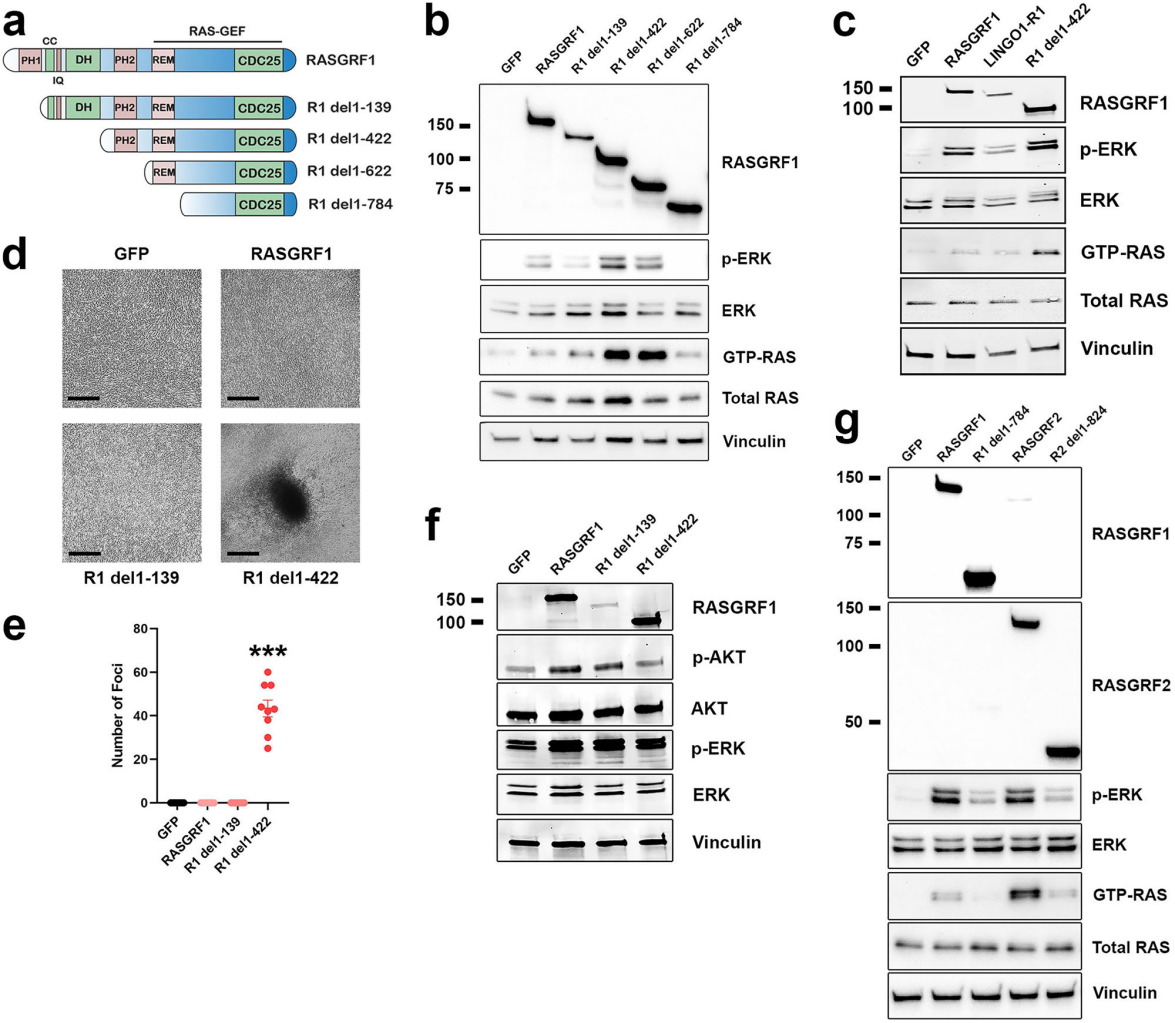
Genetic features of RASGRF fusions that contribute to RAS activation and cellular transformation. **a** Structure of full-length RASGRF1 and N-terminal truncation constructs lacking the PH1 domain (R1 del1–139), PH1 and DH domains (R1 del1–422), and constructs preserving only the REM and CDC25 domains (R1 del1–622) or only the CDC25 domain (R1 del1–784). **b** Western immunoblotting of lysates from HEK 293 T cells expressing the indicated cDNAs to assess cellular levels of active RAS (GTP-RAS) and ERK activation (p-ERK). Molecular weight is indicated in kDa on the left. **c** Western immunoblotting of lysates from HEK 293 T cells transiently transfected with the indicated cDNAs. LINGO1–R1, LINGO1–RASGRF1. **d** Tumor cell focus formation in NIH3T3 cells expressing the indicated cDNAs. Cells were cultured for 5 weeks. Images are representative of foci identified in 3 independent experiments. Black marker denotes 300 μm. **e** Quantification of NIH3T3 cell foci transduced with the indicated cDNAs after 4–6 weeks. Mean and standard error for three independent experiments (with three replicates each) are shown. ***; *P* < 0.0001 by two-tailed t-test compared to GFP. **f** Western immunoblotting of lysates from NIH3T3 cells expressing the indicated cDNAs. **g** Western immunoblotting of lysates from HEK 293 T cells transiently transfected with the indicated cDNAs.

While most RASGRF1 and RASGRF2 fusions identified in the present study lack both the PH1 and DH domains (like previously established oncogenic RASGRF1 fusions), some disrupt the PH1 domain but preserve the DH domain. To provide insight into which domains may contribute to RASGRF autoinhibition such that their absence in RASGRF fusions promotes RAS activation and cell transformation, we generated and characterized a series of RASGRF1 N-terminal deletion constructs (Fig. 6a). We compared the ability of full-length RASGRF1, RASGRF1 without the PH1 domain (R1 del1–139), RASGRF1 without the PH1 and DH domains (R1 del1–422), RASGRF1 lacking the PH1, DH, and PH2 domains with preserved REM and CDC25 domains (R1 del1–622), and RASGRF1 with only the CDC25 domain (R1 del1–784) to promote RAS activation (Fig. 6a, b). Compared to GFP, all constructs except R1 del1–784 (containing only the CDC25 domain) increased levels of GTP-RAS and MAPK signaling in HEK 293 T cells (Fig. 6b). We observed that R1 del1–422 and R1 del1–622 (with preserved REM and CDC25 domains but lacking both the PH1 and DH domains) increased RAS activation more robustly than full-length RASGRF1 or R1 del1–139 (lacking only the PH1 domain; Fig. 6b). Of note, expression of R1 del1–139 was less robust than that of the other constructs, which could contribute to its lesser degree of RAS activation.

To confirm that loss of the PH1 and DH domains of RASGRF1 promotes more robust RAS activation than loss of the PH1 domain alone, we generated one of the fusions identified in this study that lacks only the PH1 domain (LINGO1–RASGRF1; Fig. 2a). Based on the findings from Fig. 6b, we hypothesized that this fusion would promote less robust RAS activation compared to RASGRF1 lacking both the PH1 and DH domains. We transiently transfected HEK 293 T cells with GFP, RASGRF1, LINGO1–RASGRF1, or R1 del1–422 (lacking both the PH1 and DH domains). Levels of GTP-RAS and MAPK activation were similar in cells expressing RASGRF1 and LINGO1–RASGRF1 with more robust RAS activation in cells expressing R1 del1–422 (Fig. 6c). These findings are consistent with those of R1 del1–139 in Fig. 6b, suggesting that loss of the PH1 domain alone does not augment RAS activation compared to full-length RASGRF1 in our assays.

We previously demonstrated that IQGAP1–RASGRF1 (which lacks both the PH1 and DH domains and does not include an N-terminal transmembrane fusion partner) promotes proliferation with loss of contact inhibition as a marker of transformation in NIH3T3 cells^21^. We introduced GFP, full-length RASGRF1, R1 del1–139, or R1 del1–422 into NIH3T3 cells and assayed for proliferation with loss of contact inhibition. We observed that in contrast to cells expressing R1 del1–422 (lacking both the PH1 and DH domains), cells expressing full-length RASGRF1 or R1 del1–139 (without the PH1 domain) did not demonstrate loss of contact inhibition, similar to GFP (Fig. 6d, e). In contrast to our findings in HEK 293 T cells, R1 del1–422 did not clearly increase RAS activation more robustly than full-length RASGRF1 or R1 del1–139 in NIH3T3 cells (although all three constructs did increase RAS activation compared to GFP; Fig. 6f). As in HEK 293 T cells, we noted reduced overall expression of R1 del1–139 compared to the other constructs. Our findings from these assays suggest that the RASGRF fusions identified in this study, without an N-terminal transmembrane partner that lack the PH1 domain but preserve the DH domain, may have limited transforming potential and may not substantially contribute to tumorigenesis.

We note that R1 del1–784 (containing the CDC25 domain but not the REM domain) did not increase RAS activation in HEK 293 T cells (Fig. 6b). While none of the RASGRF1 fusions reported in this study lack the REM domain, 3 of the identified RASGRF2 fusions contain the CDC25 but not the REM domain (Fig. 2d). To determine if the isolated CDC25 domain of RASGRF2 can promote RAS activation, we transiently transfected HEK 293 T cells with an N-terminal deletion construct containing only the CDC25 domain of RASGRF2 (R2 del1–824) compared to full-length RASGRF2, full-length RASGRF1, and R1 del1–784. We found that the isolated CDC25 domains of both RASGRF1 and RASGRF2 promoted less RAS activation compared to their full-length counterparts (Fig. 6g). Thus, our data suggest that the CDC25 domain of RASGRF1 or RASGRF2 alone may be insufficient for RAS activation and cellular transformation unless fused to a membrane-spanning N-terminal partner (as demonstrated with OCLN–RASGRF2 v3 in Fig. 5). These findings are consistent with a prior study demonstrating ectopic expression of the CDC25 domain of RASGRF1 does not promote transformation unless targeted to the plasma membrane^28^.

## Discussion

Here, we report 40 in-frame fusions involving the catalytic RAS-GEF domain of RASGRF1 or RASGRF2 in 13 solid tumor malignancies, providing insights about the prevalence and tissue distribution of an emerging class of oncogenic fusions. Although rare, cells driven by select RASGRF1 fusions are sensitive to targeting of the downstream MAPK pathway, suggesting at least a subset of these fusions may be therapeutically actionable^6,19,21^.

Half (*n* = 20) of the fusions were identified in melanoma, PDAC, and NSCLC, which are malignancies characterized by dysregulated RAS signaling^40–43^. RASGRF fusions are enriched in otherwise driver-negative tumors in these malignancies. For example, none of the five melanomas with a RASGRF fusion identified in this study had co-occurring activating BRAF or NRAS mutations. Other RASGRF1 fusions have been described in melanocytic neoplasms with spitzoid features that lack the oncogenic HRAS mutations or kinase fusions typically found in Spitz neoplasms^20^. We identify RASGRF fusions in 7 PDACs, with only one (with an *MSH3–RASGRF2* fusion) harboring a KRAS mutation (G12D). We also previously reported an *SLC4A4–RASGRF1* fusion in a human KRAS wild-type PDAC cell line (PaCaDD137)^21^. Seven of eight NSCLC/lung cancers with a RASGRF fusion identified in this study did not have a co-occurring established oncogenic driver. However, one NSCLC harbored both an EGFR Exon 19 deletion and an *ADPGK–NTRK3* fusion in a patient previously treated with the EGFR inhibitor osimertinib. Although it is unknown whether the NTRK3 and RASGRF1 fusions were present prior to osimertinib administration, it is possible they are acquired alterations that promote osimertinb resistance. Oncogenic fusions (including NTRK1 fusions) have previously been identified as acquired mechanisms of resistance to osimertinib^44–46^. In addition, a TMEM87A–RASGRF1 fusion was shown to promote resistance to osimertinib in an EGFR-mutant NSCLC cell line^19^. We and others also previously reported RASGRF1 fusions in 2 additional lung adenocarcinomas from individuals with no smoking history without a known driver^19,21^.

A subset of RASGRF1 and RASGRF2 fusions involves N-terminal transmembrane proteins, suggesting a mechanistic role for membrane localization in fusion-mediated RAS activation. Wild-type RASGRF and SOS proteins are recruited to the membrane to facilitate RAS activation in response to upstream signaling pathways (including RTK signaling for SOS1 and calcium signaling for RASGRFs)^47,48^. We show that targeting full-length RASGRF1 or a previously reported IQGAP1–RASGRF1 fusion (that lacks membrane-spanning domains) to the plasma membrane with the addition of a C-terminal CAAX peptide promotes RAS activation and cell transformation. Conversely, disruption of plasma membrane localization of OCLN–RASGRF1 due to deletion of 5 amino acids from the 4th transmembrane domain markedly reduces RAS activation and cell transformation. However, we cannot rule out the possibility that this deletion causes global structural changes that impair catalytic activity and/or protein stability rather than simply preventing membrane localization. Overall, our findings suggest that membrane localization markedly enhances the transforming activity of RASGRF fusions, likely by facilitating nucleotide exchange of membrane-associated RAS. Of note, recurrent Rho-GAP fusions that suppress activity of the GTPase RhoA occur in diffuse-type gastric cancers^49,50^. Similar to RASGRF fusions, at least a subset of these feature transmembrane or membrane-associated N-terminal fusion partners, including some (encoded by *OCLN* and *RP2*), also identified here in RASGRF fusions^51^. These observations imply a shared mechanism of aberrant membrane localization of catalytic GEF or GAP domains to promote dysregulated RAS or Rho signaling, respectively.

The RAS-GEF domain consists of both the RAS-exchange motif (REM) domain (which binds to RAS substrates) and the CDC25 domain (which catalyzes nucleotide exchange)^29–31^. Prior studies have shown that the CDC25 domain of RASGRF1 is sufficient to promote RAS activation without the REM domain, while both the CDC25 and REM domains are required for RAS activation by SOS1^32,52,53^. While all RASGRF1 fusions identified in this study retain both the REM and CDC25 domains, 3 of the 23 identified RASGRF2 fusions preserve the CDC25 domain but not the REM domain. We demonstrate that an OCLN–RASGRF2 fusion (v3) containing only the CDC25 domain of RASGRF2 promotes RAS activation and cell transformation. In contrast, our studies of RASGRF N-terminal deletion constructs indicate that the isolated CDC25 domain without the REM domain does not substantially promote RAS signaling in the absence of membrane localization, consistent with prior studies^28^.

We find that ectopic expression of full-length RASGRF1 and RASGRF2 does not promote cellular transformation in our assays, arguing that increased expression alone may not be sufficient to promote oncogenic phenotypes. We observe that ectopic expression of a previously reported IQGAP1–RASGRF1 fusion promotes more robust RAS activation than full-length RASGRF1, suggesting that loss of functional domains within the N-terminus of RASGRF1 contributes to the oncogenic features of RASGRF1 fusions. Our study of a series of N-terminal RASGRF1 truncation constructs suggests that elimination of both the PH1 and DH domains robustly increases RAS activation and promotes cellular transformation, consistent with prior reports^29,36,37^. Oncogenic RASGRF1 fusions previously reported by us and others lack both the PH1 and DH domains^19,21^. In contrast, we find that disruption of the PH1 domain alone only mildly increases RAS activation and does not promote cellular transformation in focus formation assays in NIH3T3 cells, similar to full-length RASGRF1.

Our findings provide key insights about the genetic features of RASGRF fusions that contribute to oncogenic phenotypes. Specifically, our data suggest a model whereby RASGRF fusions containing at least the CDC25 domain fused to an N-terminal transmembrane protein that positions the catalytic CDC25 domain within the cytoplasm are sufficient for RAS activation and cellular transformation. Fusions that lack a membrane-localizing fusion partner that contain both the REM and CDC25 domain, with loss of both the PH1 and DH domains, also promote RAS activation and cellular transformation. However, our data suggest that fusions without a membrane-localizing fusion partner that either (1) contain only the CDC25 domain or (2) lack the PH1 domain while preserving the DH domain demonstrate reduced capacity for RAS activation and cellular transformation. Thus, the functional significance of the RASGRF fusions identified here without a membrane-localizing fusion partner and with a preserved DH domain (3 RASGRF1 and 10 RASGRF2 fusions) or with the CDC25 domain but not the REM domain (2 RASGRF2 fusions) is unclear. We cannot rule out the possibility that domains within some of the N-terminal fusion partners in this subset of fusions (while not membrane-spanning) could promote membrane association through alternative mechanisms to facilitate RAS activation and transformation. Of note, the only RASGRF fusion we identified in a PDAC with a concurrent KRAS mutation is MSH3-RASGRF2 (which lacks the PH1 but not DH domain), suggesting the fusion is unlikely to be an oncogenic driver in that tumor (Supplementary Fig. 2).

Both RASGRF1 and RASGRF2 activate multiple RAS isoforms (HRAS, NRAS, and KRAS)^54^. Whether they differ with regard to binding affinity for these RAS isoforms has not been clearly established. RASGRF1 and RASGRF2 have distinct tissue expression and differences in upstream activators, which may provide contextual specificity in terms of pathway regulation^29,55^. Although the studies presented here were not designed to compare functional effects of RASGRF1 and RASGRF2 fusions, we do not appreciate any gross differences in total RAS activation or cellular transformation promoted by OCLN–RASGRF1 compared to OCLN–RASGRF2 or in sensitivity of cells driven by these fusions to MEK inhibition.

The finding of recurrent RASGRF fusions in multiple malignancies raises the question of whether functional fusions involving members of the other two RAS-GEF families (SOS and RASGRP) might occur in cancer. These RAS-GEF classes share some functional domains with RASGRFs and appear to have autoinhibitory mechanisms, suggesting fusions involving other RAS-GEFs are plausible^56,57^. Further work will be necessary to determine if analogous fusions with other RAS-GEFs occur or if RASGRF1 and RASGRF2 share distinct features conducive to generating functional chimeric proteins.

## Methods

### Bioinformatic identification of RASGRF fusions and co-occurring alterations

This study was conducted with de-identified health information subject to an Institutional Review Board (IRB) exemption determination (Advarra Pro00072742) and did not involve human subjects research. Molecular and clinical data from the Tempus real-world multimodal database were de-identified in accordance with the Health Insurance Portability and Accountability Act (HIPAA) using Safe Harbor guidelines in compliance with the Declaration of Helsinki.

Patient samples subjected to Tempus xR sequencing were retrospectively identified from the Tempus real-world multimodal database and filtered to those that had a fusion involving *RASGRF1* or *RASGRF2* detected in RNA. The 639 fusions initially detected underwent rigorous filtering processes to exclude technical artifacts and to select only those fusions with sufficient evidence supporting a relevant functional effect. xR is a whole-exome capture next-generation sequencing (NGS) RNA-seq assay that quantifies transcript- and gene-level expression and identifies transcriptional evidence of chromosomal rearrangements resulting in the expression of fusion RNA species over a 39 Mb target region of the human genome (https://www.tempus.com/wp-content/uploads/2023/06/Tempus-xR_Validation.pdf)^58^. DNA was sequenced using Tempus xT, an NGS DNA panel with a tumor-normal match that detects single-nucleotide variants (SNVs), insertions and/or deletions (indels), and copy number variants (CNVs) in 596–648 genes and chromosomal rearrangements in 8–22 genes with high sensitivity and specificity^59,60^. A subset of patient samples was sequenced using Tempus xO, which interrogates alterations in 1711 genes from formalin-fixed paraffin-embedded (FFPE) tumor tissue and matched normal saliva or blood^61^.

Fusion endpoints from RNA-seq data were reviewed manually using the Integrative Genomics Viewer (https://igv.org/) to confirm exon-to-exon fusions and phase of open reading frames^62^. Prediction of location and topology of transmembrane domains in N-terminal fusion partners was performed using DeepTMHMM (10.1101/2022.04.08.487609).

### Cell lines and reagents

HEK 293 T cells were obtained from ATCC and maintained in DMEM (Gibco) with 10% heat-inactivated fetal bovine serum (FBS; Gibco) and penicillin (100 units/mL) / streptomycin (100 μg/mL; Gibco). NIH3T3 cells were obtained from ATCC and cultured in DMEM with 10% heat-inactivated fetal calf serum (Gibco) and penicillin/streptomycin (Gibco). Ba/F3 cells were kindly provided by the laboratory of Dr. Matthew Meyerson (Dana-Farber Cancer Institute) and were maintained in RPMI-1640 (Gibco) with heat-inactivated 10% FBS (Gibco), penicillin/streptomycin (Gibco), and 1 ng/mL interleukin-3 (IL3; Gibco). Ba/F3 cells expressing RASGRF fusions were cultured without IL3. Trametinib was purchased from Selleck Chemicals.

### Cloning of *RASGRF1* and *RASGRF2* cDNAs

Total RNA from PC9 cells and from PaCaDD137 cells was used as a template for RASGRF1 and RASGRF2 cDNA preparation, respectively, with oligo-dT primers using the SuperScript III First-Strand Synthesis System (Invitrogen). Full-length RASGRF1 or RASGRF2 were PCR amplified using Q5 High-Fidelity DNA Polymerase (New England BioLabs) using primers that incorporated *attB* recombination sites for Gateway cloning (Invitrogen). PCR primer sequences for cloning of all cDNAs are provided in Supplementary Table 4.

PCR products were gel-purified and cloned into pDONR223 (Invitrogen) with BP Clonase II Enzyme Mix (Invitrogen). For RASGRF1, the entry clone was then linearized using the restriction endonuclease PvuI (New England BioLabs) before sub-cloning. Constructs were sub-cloned into the lentiviral expression vector pLX307 (Addgene) with LR Clonase II Enzyme Mix (Invitrogen).

To generate cDNAs encoding RASGRF1 or IQGAP1–RASGRF1 with a C-terminal CAAX membrane localization motif, RASGRF1 or IQGAP1–RASGRF1 was amplified with a reverse primer encoding SKDGKKKKKKSKTKCVIM at the C-terminus of RASGRF1 and incorporating the *attB* recombination sites. Generation of IQGAP1–RASGRF1 was previously described^21^. RASGRF1 + CAAX and IQGAP1–RASGRF1 + CAAX cDNAs were cloned into pDONR223, followed by pLX307 as above. Amino acids 255-259 were deleted from OCLN–RASGRF1 in pDONR223 using the Q5 Site-Directed Mutagenesis kit (New England BioLabs) to generate OCLN–RASGRF1 del255–259. The cDNA was then subcloned into pLX307 as above.

OCLN–RASGRF2 v1 and v3 fusions were generated with Gene Splicing by Overlap Extension PCR using OCLN–RASGRF1 and RASGRF2 cDNA as template^63^. Generation of OCLN–RASGRF1 was previously described^21^. The OCLN and RASGRF2 portions of each fusion were amplified from OCLN–RASGRF1 and RASGRF2, respectively. PCR products were gel-purified, and equimolar amounts of overlapping PCR products to generate OCLN–RASGRF2 v1 or v3 were used as templates for a secondary PCR using primers with *attB* recombination sites. Full-length OCLN–RASGRF2 v1 or v3 PCR products were cloned into pDONR223, followed by pLX307 as above.

RASGRF1 N-terminal truncation variants were generated using full-length RASGRF1 as a template for PCR and primers incorporating *attB* recombination sites. R1 del1–139 (lacking the PH1 domain), R1 del1–422 (lacking the PH1 and DH domains), and R1 del1–784 (containing only the CDC25 domain) utilized endogenous methionine residues as start codons. An ATG start codon was incorporated into the forward PCR primer used to generate R1 del1–622 (containing only the REM and CDC25 domains). The RASGRF2 N-terminal truncation variant R2 del1-824 (containing only the CDC25 domain) was generated using full-length RASGRF2 as a template for PCR and primers incorporating *attB* recombination sites. An ATG start codon was incorporated into the forward PCR primer. The LINGO1–RASGRF1 fusion (which contains only the first two amino acids of LINGO1) was generated using full-length RASGRF1 as a template for PCR. The forward PCR primer used to generate the fusion included the coding sequence for the first two amino acids of LINGO1 (ATGCAG) and the 5’ portion of Exon 2 of RASGRF1 present in the fusion. All PCR products were cloned into pDONR223 followed by pLX307 as above.

All cDNA clones were confirmed by Sanger sequencing. Lentivirus was produced as previously described^64^.

### RAS activation assay

HEK 293 T cells were transduced with lentivirus carrying plasmid cDNAs in the presence of 4 μg/mL polybrene. Cells were centrifuged at 2250 rpm for 30 min. The following day, transduced cells were selected with 1 μg/mL puromycin for at least 5 days. For assays performed with transiently transfected cells, plasmid DNA was complexed with Polyethylenimine (PEI 25 K; Kyfora Bio) for 20 min in OptiMEM at a ratio of 1 μg plasmid DNA to 4 μg PEI. The complex was then used to transfect HEK 293 T cells for 16–24 h. Cell lysates were collected and subjected to RAS activation assays using the Active RAS Detection Kit (Cell Signaling Technology #8821) according to the manufacturer’s instructions.

### Confocal microscopy

Assayed cDNAs were cloned into the Gateway pcDNA-DEST53 vector containing an N-terminal GFP tag (Invitrogen #12288015) using LR Clonase II Enzyme Mix. HEK293T cells were seeded into 96-well glass-bottom tissue culture plates (Cellvis) coated with poly-d-lysine (Gibco). Twenty-four hours later, cells were transfected with plasmid cDNA using Lipofectamine 3000 Reagent (Invitrogen #L3000001) according to the manufacturer’s protocol. Twenty-four hours after transfection, cells were stained with CellMask Deep Red Plasma Membrane stain (Invitrogen #C10046) according to the manufacturer’s instructions. Images were captured with a Zeiss LSM 880 Airyscan confocal microscope with a 63× oil immersion objective.

### Anchorage-independent proliferation and focus formation assays

NIH3T3 cells were transduced with lentivirus carrying plasmid cDNAs in the presence of 4 μg/mL polybrene. Cells were centrifuged at 2250 rpm for 30 min. The following day, transduced cells were selected with 0.75 μg/mL puromycin for 2–5 days. For anchorage-independent proliferation assays, cells were plated in triplicate in growth media plus 0.4% agar on top of a layer of growth media plus 0.8% agar in a 6-well tissue culture plate. Cells were cultured in agar for 15–21 days with fresh media added weekly. Images were captured using a BioTek Cytation 5 Cell Imaging Multimode Reader (Agilent Technologies) with a 10× phase contrast objective. The colony area was quantified using ImageJ analysis software. For focus formation assays, cells were plated in triplicate in a 6-well tissue culture plate. Cells were cultured for 4–6 weeks with fresh media added every 3 days. Images were captured as above for anchorage-independent proliferation assays. Statistical analysis was performed with GraphPad Prism 9 software.

### Ba/F3 transformation and cell viability assays

Assayed cDNAs were introduced into Ba/F3 cells via lentiviral transduction with 8 μg/mL polybrene. Cells were centrifuged at 2250 rpm for 30 min. The following day, transduced cells were selected with 1 μg/mL puromycin for at least 5 days. IL3 was then withdrawn from culture media to assess IL3-independent proliferation.

For cell viability assays, Ba/F3 cells were seeded in white, clear-bottom 96-well microtiter plates. Trametinib or the drug vehicle was added on the same day. After 4 days of drug exposure, cell viability was determined using CellTiter-Glo (Promega) according to the manufacturer’s instructions. Luminescence was measured with a Spectramax M3 plate reader (Molecular Devices). Data analysis was performed using GraphPad Prism software.

### Antibodies and Immunoblotting

Antibody against RASGRF1 was obtained from Abcam (#ab111830). Antibody against RASGRF2 was obtained from Thermo Scientific (#PA-28867). Antibodies against AKT (#2920), phospho-AKT (Ser 473; #4060), ERK 1/2 (#4695), phospho-ERK (Thr 202/Tyr 204; #9101), occludin (#91131), and cleaved PARP (#9548) were obtained from Cell Signaling Technology. Antibody against vinculin (#V9131) was obtained from Sigma.

Cell lysates were prepared with RIPA buffer (Abcam #ab156034) supplemented with protease inhibitors (Roche #11836153001) and phosphatase inhibitors (Sigma #P5726, #P0044). Lysates were fractionated by SDS-PAGE and transferred to nitrocellulose membranes using the Trans-Blot Turbo transfer system (Bio-Rad). Chemiluminescence was performed using SuperSignal West Pico Plus or Femto Chemiluminescent Substrate (Thermo Scientific #34580, #34096). HRP-conjugated anti-mouse and anti-rabbit secondary antibodies were obtained from Cytiva (#NA931, #NA934). Image acquisition was performed with a ChemiDoc MP Imaging system (Bio-Rad). For some experiments, two-color immunoblotting was performed with fluorescence detection using an Odyssey M imager (LICORbio). For these studies, goat anti-mouse IgG (H&L) secondary antibody (DyLight 800 4× PEG) and goat anti-rabbit IgG (H&L) highly cross-adsorbed secondary antibody (Alexa Fluor 680) were utilized (Invitrogen #SA5-35521, #A-21109).

### Statistical analysis

Data were plotted as mean ± SEM from at least three independent experiments as indicated in the figure legends. Statistical analysis was performed with an unpaired two-tailed Student’s *t* test using GraphPad Prism (version 9). *P* < 0.05 was considered significant.

## Supplementary information

Supplementary Data v2 submission

Supplementary Data Table 4

## Data Availability

Some of the data included in this work were collected in a real-world health care setting and are subject to controlled access for privacy and proprietary reasons. When possible, de-identified derived data supporting the findings of this study have been made available within this published article and its supplementary information files. Requests for materials generated from this study should be directed to the corresponding author and will be evaluated in accordance with relevant policies and procedures.

## References

[1] Cox, A. D., Fesik, S. W., Kimmelman, A. C., Luo, J. & Der, C. J. Drugging the undruggable RAS: Mission possible?. *Nat. Rev. Drug Discov.* **13**, 828–851 (2014). 25323927 10.1038/nrd4389 PMC4355017

[2] Prior, I. A., Hood, F. E. & Hartley, J. L. The frequency of ras mutations in cancer. *Cancer Res.* **80**, 2969–2974 (2020). 32209560 10.1158/0008-5472.CAN-19-3682 PMC7367715

[3] Lemmon, M. A. & Schlessinger, J. Cell signaling by receptor tyrosine kinases. *Cell* **141**, 1117–1134 (2010). 20602996 10.1016/j.cell.2010.06.011 PMC2914105

[4] Vogelstein, B. et al. Cancer genome landscapes. *Science* **339**, 1546–1558 (2013). 23539594 10.1126/science.1235122 PMC3749880

[5] Simanshu, D. K., Nissley, D. V. & McCormick, F. RAS Proteins and Their Regulators in Human Disease. *Cell* **170**, 17–33 (2017). 28666118 10.1016/j.cell.2017.06.009 PMC5555610

[6] Hayashi, T. et al. RASA1 and NF1 are preferentially co-mutated and define a distinct genetic subset of smoking-associated non-small cell lung carcinomas sensitive to MEK inhibition. *Clin. Cancer Res.* **24**, 1436–1447 (2018). 29127119 10.1158/1078-0432.CCR-17-2343 PMC6440215

[7] Mertens, F., Johansson, B., Fioretos, T. & Mitelman, F. The emerging complexity of gene fusions in cancer. *Nat. Rev. Cancer* **15**, 371–381 (2015). 25998716 10.1038/nrc3947

[8] Tuna, M., Amos, C. I. & Mills, G. B. Molecular mechanisms and pathobiology of oncogenic fusion transcripts in epithelial tumors. *Oncotarget* **10**, 2095–2111 (2019). 31007851 10.18632/oncotarget.26777 PMC6459343

[9] Fernandez-Cuesta, L. et al. CD74-NRG1 fusions in lung adenocarcinoma. *Cancer Discov.* **4**, 415–422 (2014). 24469108 10.1158/2159-8290.CD-13-0633

[10] Ou, S. I. et al. Identification of Novel CDH1-NRG2α and F11R-NRG2α fusions in NSCLC plus additional novel NRG2α fusions in other solid tumors by whole transcriptome sequencing. *JTO Clin. Res. Rep.* **2**, 100132 (2021). 34589990 10.1016/j.jtocrr.2020.100132 PMC8474258

[11] Ross, J. S. et al. The distribution of BRAF gene fusions in solid tumors and response to targeted therapy. *Int. J. Cancer* **138**, 881–890 (2016). 26314551 10.1002/ijc.29825 PMC5049644

[12] Tomlins, S. A. et al. Recurrent fusion of TMPRSS2 and ETS transcription factor genes in prostate cancer. *Science* **310**, 644–648 (2005). 16254181 10.1126/science.1117679

[13] Tonon, G. et al. t(11;19)(q21;p13) translocation in mucoepidermoid carcinoma creates a novel fusion product that disrupts a Notch signaling pathway. *Nat. Genet.* **33**, 208–213 (2003). 12539049 10.1038/ng1083

[14] French, C. A. et al. Midline carcinoma of children and young adults with NUT rearrangement. *J. Clin. Oncol.* **22**, 4135–4139 (2004). 15483023 10.1200/JCO.2004.02.107

[15] Izumi, H. et al. The CLIP1-LTK fusion is an oncogenic driver in non-small-cell lung cancer. *Nature* **600**, 319–323 (2021). 34819663 10.1038/s41586-021-04135-5 PMC8687755

[16] Drilon, A. et al. Response to ERBB3-directed targeted therapy in NRG1-rearranged cancers. *Cancer Discov.* **8**, 686–695 (2018). 29610121 10.1158/2159-8290.CD-17-1004 PMC5984717

[17] Schram, A. M. et al. Zenocutuzumab, a HER2xHER3 bispecific antibody, is effective therapy for tumors driven by NRG1 gene rearrangements. *Cancer Discov.* **12**, 1233–1247 (2022). 35135829 10.1158/2159-8290.CD-21-1119 PMC9394398

[18] Schram, A. M. et al. Efficacy of zenocutuzumab in NRG1 fusion-positive cancer. *N. Engl. J. Med.* **392**, 566–576 (2025). 39908431 10.1056/NEJMoa2405008 PMC11878197

[19] Cooper, A. J. et al. Identification of a RAS-activating TMEM87A-RASGRF1 fusion in an exceptional responder to sunitinib with non-small cell lung cancer. *Clin Cancer Res.* **26**, 4072–4079 (2020). 32312893 10.1158/1078-0432.CCR-20-0397 PMC7415568

[20] Goto, K. et al. RASGRF1-rearranged cutaneous melanocytic neoplasms with spitzoid cytomorphology: a clinicopathologic and genetic study of 3 cases. *Am. J. Surg. Pathol.* **46**, 655–663 (2022). 34799483 10.1097/PAS.0000000000001839

[21] Hunihan, L. et al. RASGRF1 fusions activate oncogenic RAS signaling and confer sensitivity to MEK inhibition. *Clin. Cancer Res.* **28**, 3091–3103 (2022). 35247929 10.1158/1078-0432.CCR-21-4291 PMC9288503

[22] Houlier, A. et al. RASGRF2 gene fusions identified in a variety of melanocytic lesions with distinct morphological features. *Pigment Cell Melanoma Res.* **34**, 1074–1083 (2021). 34310073 10.1111/pcmr.13004

[23] Watts, J. M. et al. A Case of AML Characterized by a Novel t(4;15)(q31;q22) translocation that confers a growth-stimulatory response to retinoid-based therapy. *Int. J. Mol. Sci.* **18**, 1492 (2017). 28696354 10.3390/ijms18071492 PMC5535982

[24] Colombino, M. et al. BRAF/NRAS mutation frequencies among primary tumors and metastases in patients with melanoma. *J. Clin. Oncol.* **30**, 2522–2529 (2012). 22614978 10.1200/JCO.2011.41.2452

[25] Hodis, E. et al. A landscape of driver mutations in melanoma. *Cell* **150**, 251–263 (2012). 22817889 10.1016/j.cell.2012.06.024 PMC3600117

[26] Witkiewicz, A. K. et al. Whole-exome sequencing of pancreatic cancer defines genetic diversity and therapeutic targets. *Nat. Commun.* **6**, 6744 (2015). 25855536 10.1038/ncomms7744 PMC4403382

[27] Subramanian, V. S., Marchant, J. S., Ye, D., Ma, T. Y. & Said, H. M. Tight junction targeting and intracellular trafficking of occludin in polarized epithelial cells. *Am. J. Physiol. Cell Physiol.* **293**, C1717–C1726 (2007). 17855770 10.1152/ajpcell.00309.2007

[28] Quilliam, L. A. et al. Membrane-targeting potentiates guanine nucleotide exchange factor CDC25 and SOS1 activation of Ras transforming activity. *Proc. Natl Acad. Sci. USA* **91**, 8512–8516 (1994). 8078913 10.1073/pnas.91.18.8512 PMC44636

[29] Fernández-Medarde, A. & Santos, E. The RasGrf family of mammalian guanine nucleotide exchange factors. *Biochim. Biophys. Acta* **1815**, 170–188 (2011). 21111786 10.1016/j.bbcan.2010.11.001

[30] Lai, C. C., Boguski, M., Broek, D. & Powers, S. Influence of guanine nucleotides on complex formation between Ras and CDC25 proteins. *Mol. Cell Biol.* **13**, 1345–1352 (1993). 8441380 10.1128/mcb.13.3.1345-1352.1993 PMC359443

[31] Coccetti, P., Mauri, I., Alberghina, L., Martegani, E. & Parmeggiani, A. The minimal active domain of the mouse ras exchange factor CDC25Mm. *Biochem. Biophys. Res Commun.* **206**, 253–259 (1995). 7818528 10.1006/bbrc.1995.1035

[32] Sondermann, H. et al. Structural analysis of autoinhibition in the Ras activator Son of sevenless. *Cell* **119**, 393–405 (2004). 15507210 10.1016/j.cell.2004.10.005

[33] Tartaglia, M. et al. Gain-of-function SOS1 mutations cause a distinctive form of Noonan syndrome. *Nat. Genet.* **39**, 75–79 (2007). 17143282 10.1038/ng1939

[34] Gureasko, J. et al. Role of the histone domain in the autoinhibition and activation of the Ras activator Son of Sevenless. *Proc. Natl Acad. Sci. USA* **107**, 3430–3435 (2010). 20133692 10.1073/pnas.0913915107 PMC2816639

[35] Lepri, F. et al. SOS1 mutations in Noonan syndrome: molecular spectrum, structural insights on pathogenic effects, and genotype-phenotype correlations. *Hum. Mutat.* **32**, 760–772 (2011). 21387466 10.1002/humu.21492 PMC3118925

[36] Baouz, S., Jacquet, E., Bernardi, A. & Parmeggiani, A. The N-terminal moiety of CDC25(Mm), a GDP/GTP exchange factor of Ras proteins, controls the activity of the catalytic domain. Modulation by calmodulin and calpain. *J. Biol. Chem.* **272**, 6671–6676 (1997). 9045698 10.1074/jbc.272.10.6671

[37] Cen, H., Papageorge, A. G., Vass, W. C., Zhang, K. E. & Lowy, D. R. Regulated and constitutive activity by CDC25Mm (GRF), a Ras-specific exchange factor. *Mol. Cell Biol.* **13**, 7718–7724 (1993). 8246988 10.1128/mcb.13.12.7718-7724.1993 PMC364843

[38] Arozarena, I. et al. The Rho family GTPase Cdc42 regulates the activation of Ras/MAP kinase by the exchange factor Ras-GRF. *J. Biol. Chem.* **275**, 26441–26448 (2000). 10840034 10.1074/jbc.M002992200

[39] Freshney, N. W., Goonesekera, S. D. & Feig, L. A. Activation of the exchange factor Ras-GRF by calcium requires an intact Dbl homology domain. *FEBS Lett.* **407**, 111–115 (1997). 9141492 10.1016/s0014-5793(97)00309-8

[40] Chang, M. T. et al. Identifying recurrent mutations in cancer reveals widespread lineage diversity and mutational specificity. *Nat. Biotechnol.* **34**, 155–163 (2016). 26619011 10.1038/nbt.3391 PMC4744099

[41] Ding, L. et al. Somatic mutations affect key pathways in lung adenocarcinoma. *Nature* **455**, 1069–1075 (2008). 18948947 10.1038/nature07423 PMC2694412

[42] Hayward, N. K. et al. Whole-genome landscapes of major melanoma subtypes. *Nature* **545**, 175–180 (2017). 28467829 10.1038/nature22071

[43] Bailey, P. et al. Genomic analyses identify molecular subtypes of pancreatic cancer. *Nature* **531**, 47–52 (2016). 26909576 10.1038/nature16965

[44] Lin, G., Liu, Y., Li, H., Chen, S. & Guo, Y. Emergence of NOTCH2-NTRK1 after osimertinib in a patient with lung adenocarcinoma with neuroendocrine differentiation. *Clin. Lung Cancer* **22**, e712–e715 (2021). 33714692 10.1016/j.cllc.2021.01.015

[45] Wang, J. L. et al. Survival benefit of combinatorial osimertinib rechallenge and entrectinib in an EGFR-mutant NSCLC patient with acquired LMNA-NTRK1 fusion following osimertinib resistance. *Respirol. Case Rep.* **10**, e01054 (2022). 36258694 10.1002/rcr2.1054 PMC9574602

[46] Chmielecki, J. et al. Analysis of acquired resistance mechanisms to osimertinib in patients with EGFR-mutated advanced non-small cell lung cancer from the AURA3 trial. *Nat. Commun.* **14**, 1071 (2023). 36849516 10.1038/s41467-023-35962-x PMC9971022

[47] Gureasko, J. et al. Membrane-dependent signal integration by the Ras activator Son of sevenless. *Nat. Struct. Mol. Biol.* **15**, 452–461 (2008). 18454158 10.1038/nsmb.1418 PMC2440660

[48] Farnsworth, C. L. et al. Calcium activation of Ras mediated by neuronal exchange factor Ras-GRF. *Nature* **376**, 524–527 (1995). 7637786 10.1038/376524a0

[49] Cancer Genome Atlas Research Network. Comprehensive molecular characterization of gastric adenocarcinoma. *Nature* **513**, 202–209 (2014). 10.1038/nature13480 PMC4170219 25079317

[50] Yang, H. et al. RhoGAP domain-containing fusions and PPAPDC1A fusions are recurrent and prognostic in diffuse gastric cancer. *Nat. Commun.* **9**, 4439 (2018). 30361512 10.1038/s41467-018-06747-4 PMC6202325

[51] Komatsu, M. et al. ARHGAP-RhoA signaling provokes homotypic adhesion-triggered cell death of metastasized diffuse-type gastric cancer. *Oncogene* **41**, 4779–4794 (2022). 36127398 10.1038/s41388-022-02469-6

[52] Margarit, S. M. et al. Structural evidence for feedback activation by Ras.GTP of the Ras-specific nucleotide exchange factor SOS. *Cell* **112**, 685–695 (2003). 12628188 10.1016/s0092-8674(03)00149-1

[53] Freedman, T. S. et al. A Ras-induced conformational switch in the Ras activator Son of sevenless. *Proc. Natl. Acad. Sci. USA* **103**, 16692–16697 (2006). 17075039 10.1073/pnas.0608127103 PMC1629002

[54] Wang, Q., Siminovitch, K. A., Downey, G. P. & McCulloch, C. A. Ras-guanine-nucleotide-releasing factors 1 and 2 interact with PLCγ at focal adhesions to enable IL-1-induced Ca(2+) signalling, ERK activation and MMP-3 expression. *Biochem. J.* **449**, 771–782 (2013). 23145787 10.1042/BJ20121170

[55] Feig, L. A. Regulation of neuronal function by Ras-GRF exchange factors. *Genes Cancer* **2**, 306–319 (2011). 21779501 10.1177/1947601911408077 PMC3128633

[56] Mitin, N., Rossman, K. L. & Der, C. J. Signaling interplay in Ras superfamily function. *Curr. Biol.* **15**, R563–R574 (2005). 16051167 10.1016/j.cub.2005.07.010

[57] Iwig, J. S. et al. Structural analysis of autoinhibition in the Ras-specific exchange factor RasGRP1. *Elife* **2**, e00813 (2013). 23908768 10.7554/eLife.00813 PMC3728621

[58] Michuda, J. et al. Validation of a transcriptome-based assay for classifying cancers of unknown primary origin. *Mol. Diagn. Ther.* **27**, 499–511 (2023). 37099070 10.1007/s40291-023-00650-5 PMC10300170

[59] Beaubier, N. et al. Integrated genomic profiling expands clinical options for patients with cancer. *Nat. Biotechnol.* **37**, 1351–1360 (2019). 31570899 10.1038/s41587-019-0259-z

[60] Beaubier, N. et al. Clinical validation of the tempus xT next-generation targeted oncology sequencing assay. *Oncotarget* **10**, 2384–2396 (2019). 31040929 10.18632/oncotarget.26797 PMC6481324

[61] Beaubier, N. et al. Clinical validation of the Tempus xO assay. *Oncotarget* **9**, 25826–25832 (2018). 29899824 10.18632/oncotarget.25381 PMC5995233

[62] Robinson, J. T. et al. Integrative genomics viewer. *Nat. Biotechnol.* **29**, 24–26 (2011). 21221095 10.1038/nbt.1754 PMC3346182

[63] Horton, R. M., Cai, Z. L., Ho, S. N. & Pease, L. R. Gene splicing by overlap extension: tailor-made genes using the polymerase chain reaction. *Biotechniques* **8**, 528–535 (1990). 2357375

[64] Kryukov, G. V., et al. MTAP deletion confers enhanced dependency on the PRMT5 arginine methyltransferase in cancer cells. *Science* **351**, 1214–1218 (2016). 26912360 10.1126/science.aad5214 PMC4997612

