# Supplementary material for: Genetic and oncogenic features of RASGRF fusions
Source: NPJ Precis Oncol. 2025 Jul 5;9:224. doi: 10.1038/s41698-025-01017-1 (PMC12227644; doi:10.1038/s41698-025-01017-1)
Supplement: Supplementary file 1 — Supplementary Data v2 submission [file 41698_2025_1017_MOESM1_ESM.docx]

**SUPPLEMENTARY DATA**

|  | **Supplementary Table 1: Genomic Coordinates and Number of Reads Supporting RASGRF Fusions of Unknown Significance Without a Preserved Reading Frame** | | | | | |  |
| --- | --- | --- | --- | --- | --- | --- | --- |
|  | **Fusion Pair** | **Tumor Type** | **Reference Genome** | **5' Position** | **3' Position** | **Read Support** |  |
|  | IGF1R-RASGRF1 | Breast | GRCh37 | 15:99251336 | 15:79356868 | 91 |  |
|  | RMND1-RASGRF1 | Breast | GRCh38 | 6:151417279 | 15:79058481 | 135 |  |
|  | FAM193B-RASGRF2 | Connective Tissue | GRCh38 | 5:177532443 | 5:81113538 | 19 |  |
|  | ERBIN-RASGRF2 | Melanoma | GRCh38 | 5:65992907 | 5:81113538 | 22 |  |
|  | DCDC1-RASGRF2 | Breast | GRCh38 | 11:31064469 | 5:81180175 | 8 |  |
|  | MSH3-RASGRF2 | Brain | GRCh37 | 5:80109560 | 5:80338696 | 10 |  |
|  |  |  |  |  |  |  |  |

| **Supplementary Table 2: Genomic Coordinates and Number of Reads Supporting RASGRF Fusions** | | | | | |
| --- | --- | --- | --- | --- | --- |
| **Fusion Pair** | **Tumor Type** | **Reference Genome** | **5' Position** | **3' Position** | **Read Support** |
| CMTM4-RASGRF1 | Colorectal | GRCh37 | 16:66655965 | 15:79320201 | 31 |
| DLG1-RASGRF1 | NSCLC | GRCh38 | 3:197282679 | 15:79020104 | 69 |
| FAM174B-RASGRF1 | CT | GRCh37 | 15:93173444 | 15:79320201 | 67 |
| IL6R-RASGRF1 | Unknown site | GRCh37 | 1:154427057 | 15:79320201 | 201 |
| IQGAP1-RASGRF1 | Melanoma | GRCh37 | 15:91038067 | 15:79320201 | 215 |
| LINGO1-RASGRF1 | Breast | GRCh37 | 15:77924652 | 15:79356868 | 35 |
| MAP2K5-RASGRF1 | Esophagus/Stomach | GRCh37 | 15:67923265 | 15:79356868 | 17 |
| NEO1-RASGRF1 | Pancreas | GRCh37 | 15:73575452 | 15:79320201 | 26 |
| NPTN-RASGRF1 | NSCLC | GRCh37 | 15:73855577 | 15:79298776 | 70 |
| OCLN-RASGRF1 | Cholangiocarcinoma | GRCh37 | 5:68830666 | 15:79320201 | 39 |
| PACSIN2-RASGRF1 | NSCLC | GRCh37 | 22:43275054 | 15:79320201 | 118 |
| PIAS1-RASGRF1 | NSCLC | GRCh37 | 15:68439038 | 15:79341930 | 13 |
| PRKG2-RASGRF1 | Melanoma | GRCh38 | 4:81204587 | 15:79027859 | 93 |
| RP2-RASGRF1 | NSCLC | GRCh38 | X:46837202 | 15:79020104 | 49 |
| SMAD3-RASGRF1 | Bladder | GRCh37 | 15:67358698 | 15:79320201 | 206 |
| TICRR-RASGRF1 | Melanoma | GRCh37 | 15:90129173 | 15:79324658 | 15 |
| TMEM87A-RASGRF1 | Esophagus/Stomach | GRCh37 | 15:42519004 | 15:79320201 | 150 |
| ERBIN-RASGRF2 | Melanoma | GRCh38 | 5:65992907 | 5:81108996 | 39 |
| FARP1-RASGRF2 | Cholangiocarcinoma | GRCh38 | 13:98390880 | 5:81112610 | 16 |
| FARP1-RASGRF2 | Pancreas | GRCh38 | 13:98393718 | 5:81108996 | 6 |
| GPM6B-RASGRF2 | Melanoma | GRCh37 | X:13794357 | 5:80366311 | 271 |
| HSPA4-RASGRF2 | Lung | GRCh37 | 5:132406188 | 5:80338696 | 10 |
| MRPL23-RASGRF2 | Prostate | GRCh37 | 11:1974085 | 5:80381621 | 34 |
| MSH3-RASGRF2 | Rectum | GRCh37 | 5:80064822 | 5:80338696 | 18 |
| MSH3-RASGRF2 | Ovarian | GRCh37 | 5:80088663 | 5:80338696 | 16 |
| MSH3-RASGRF2 | Pancreas | GRCh37 | 5:80057497 | 5:80338696 | 10 |
| MSH3-RASGRF2 | Head and Neck | GRCh37 | 5:79961182 | 5:80338696 | 11 |
| MSH3-RASGRF2 | Prostate | GRCh37 | 5:79974912 | 5:80363851 | 10 |
| MSH3-RASGRF2 | Prostate | GRCh37 | 5:80150135 | 5:80338696 | 15 |
| MTREX-RASGRF2 | Prostate | GRCh38 | 5:55344620 | 5:81201330 | 21 |
| NGLY1-RASGRF2 | Prostate | GRCh37 | 3:25770624 | 5:80338696 | 48 |
| OCLN-RASGRF2 | Pancreas | GRCh38 | 5:69534839 | 5:81206845 | 78 |
| OCLN-RASGRF2 | NSCLC | GRCh38 | 5:69514109 | 5:81108996 | 44 |
| OCLN-RASGRF2 | Pancreas | GRCh38 | 5:69514109 | 5:81108996 | 41 |
| OCLN-RASGRF2 | Pancreas | GRCh38 | 5:69534839 | 5:81113538 | 14 |
| OCLN-RASGRF2 | Pancreas | GRCh38 | 5:69514109 | 5:81108996 | 6 |
| SERINC5-RASGRF2 | Brain | GRCh38 | 5:80255896 | 5:81042877 | 6 |
| SKIC3-RASGRF2 | Breast | GRCh38 | 5:95512477 | 5:81180175 | 17 |
| SLC4A4-RASGRF2 | Ovarian | GRCh38 | 4:71560254 | 5:81108996 | 34 |
| TOM1L2-RASGRF2 | NSCLC | GRCh37 | 17:17764790 | 5:80338696 | 11 |

| **Supplementary Table 3: Smoking Status by Cancer Type** | | | |  |  |
| --- | --- | --- | --- | --- | --- |
| **Smoking Status** | **Melanoma (n=5)** | **NSCLC / Lung (n=8)** | **Pancreas (n=7)** | **Prostate (n=5)** | **Other (n=15)** |
| Never-Smoker | 1 (33%) | 2 (25%) | 0 (0%) | 0 (0%) | 5 (42%) |
| Current / Former | 2 (66%) | 6 (75%) | 3 (100%) | 4 (100%) | 7 (58%) |
| Unknown | 2 | 0 | 4 | 1 | 3 |


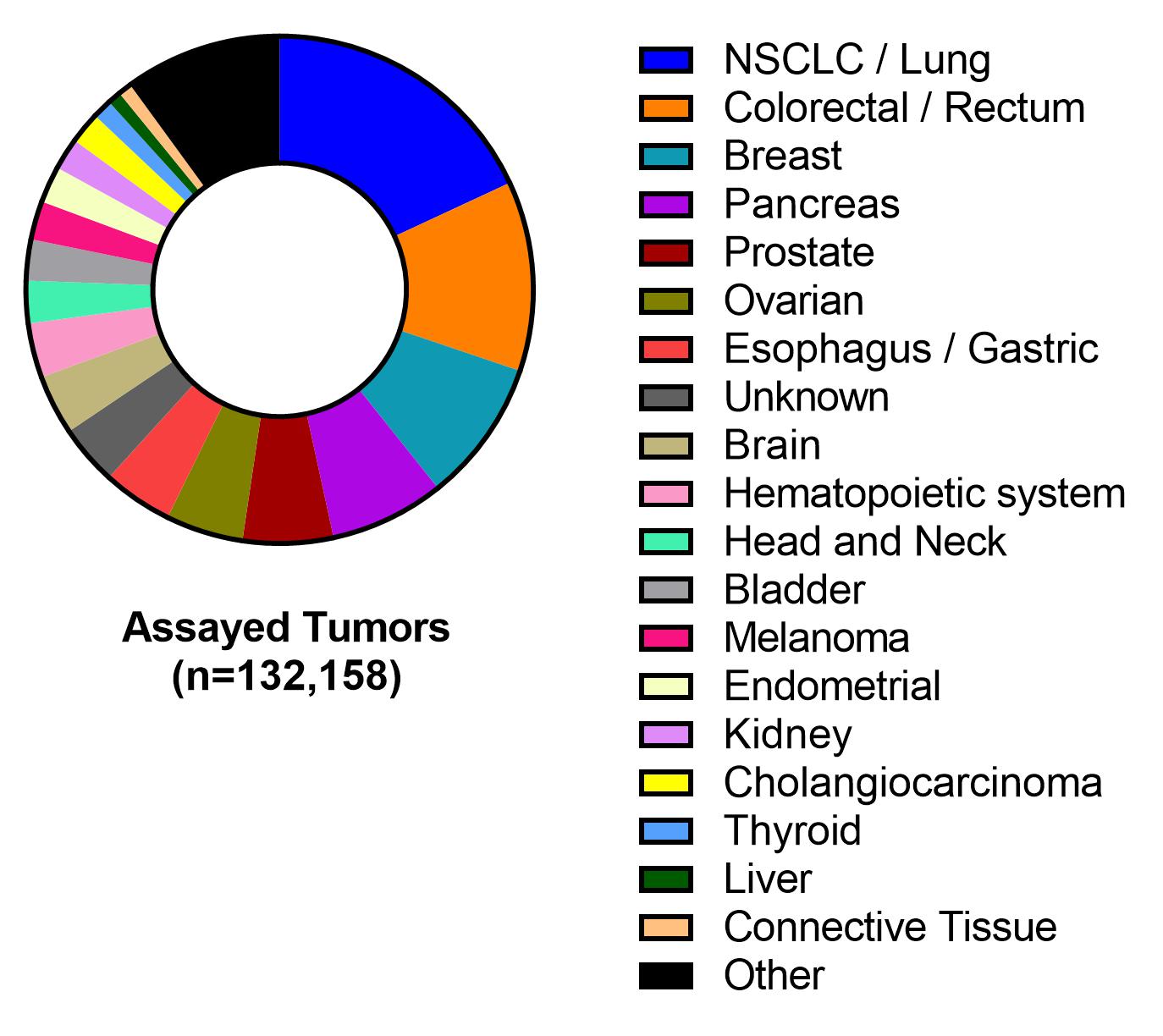


**Supplementary Figure 1.** Frequency of tumor types represented among the 132,158 tumors queried for RASGRF fusions.

**
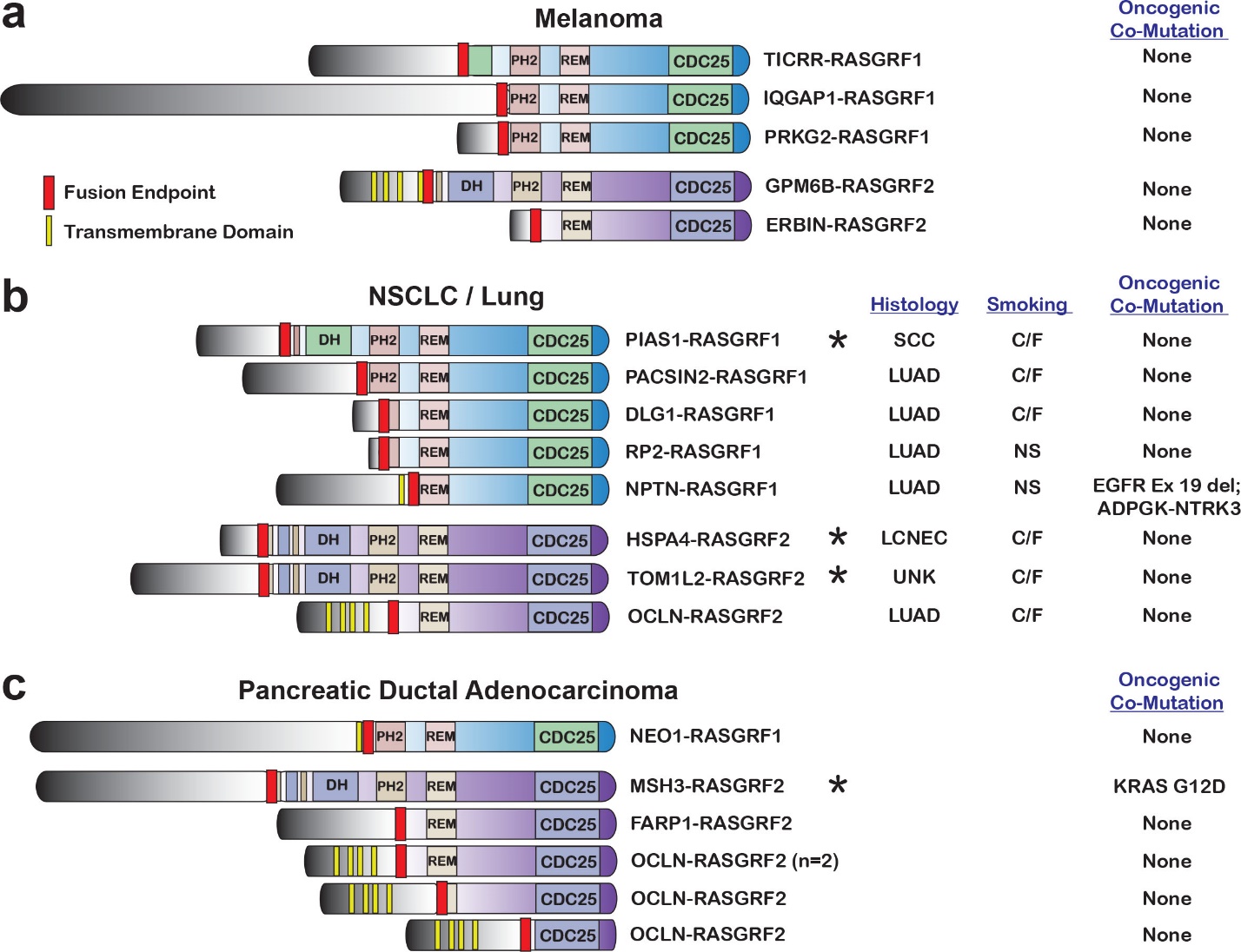
**

**Supplementary Figure 2.** RASGRF fusions identified in select malignancies. **A.** RASGRF fusions identified from melanomas. No co-occurring established oncogenic alterations were identified. DH, dbl-homology region; PH2, pleckstrin homology domain 2; REM, Ras-exchanger stabilization motif domain. **B.** RASGRF fusions identified from NSCLC / lung cancers. Asterisks denote fusions that lack transmembrane domains and feature loss of the pleckstrin homology 1 (PH1) domain but preservation of the DH domain. Functional studies in Fig. 6 indicate these fusions are unlikely to be transforming. Co-occurring oncogenic alterations were identified in one tumor with an *NPTN-RASGRF1* fusion from a patient previously treated with osimertinib. Tumor histology and patient smoking status are indicated. SCC, squamous cell carcinoma; LUAD, lung adenocarcinoma; LCNEC, large cell neuroendocrine carcinoma; UNK, unknown; C/F, current / former smoker; NS, never-smoker. **C.** RASGRF fusions identified from pancreatic ductal adenocarcinomas. A co-occurring oncogenic alteration (KRAS G12D) was identified in one tumor with an *MSH3-RASGRF2* fusion. Of note, the fusion identified in this tumor is unlikely to be transforming based on data shown in Fig. 6.

**
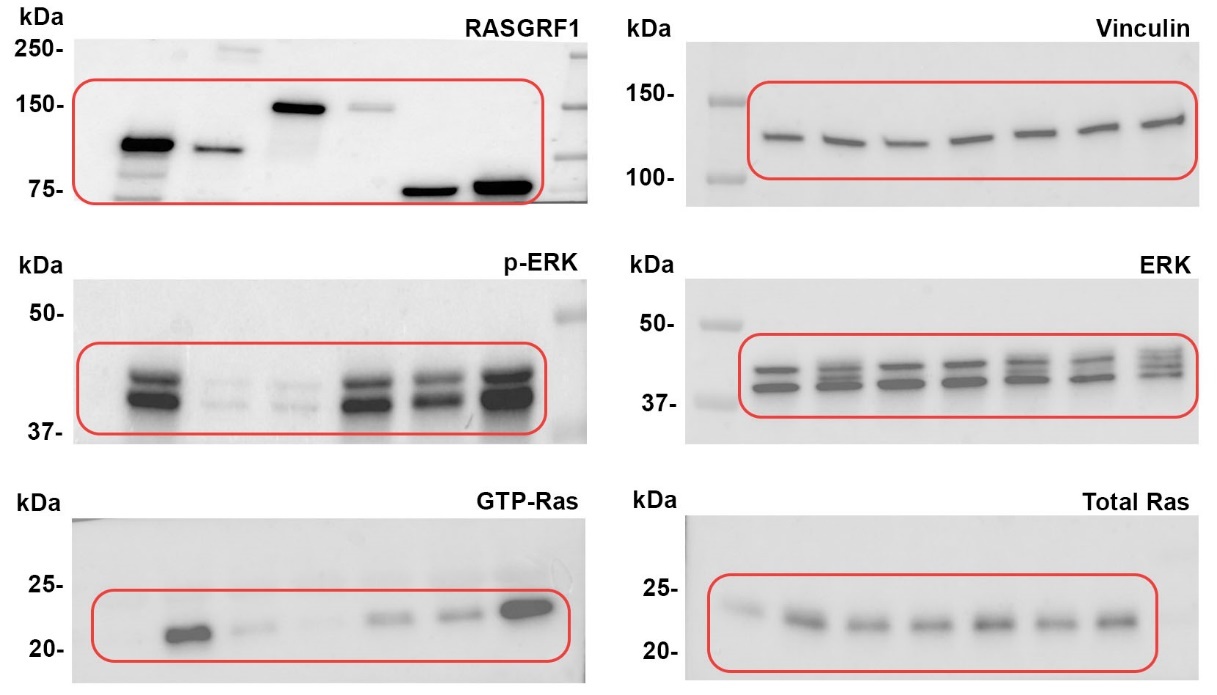
**

**Supplementary Figure 3.** Uncropped images corresponding to Western blot shown in Figure 4b.

**
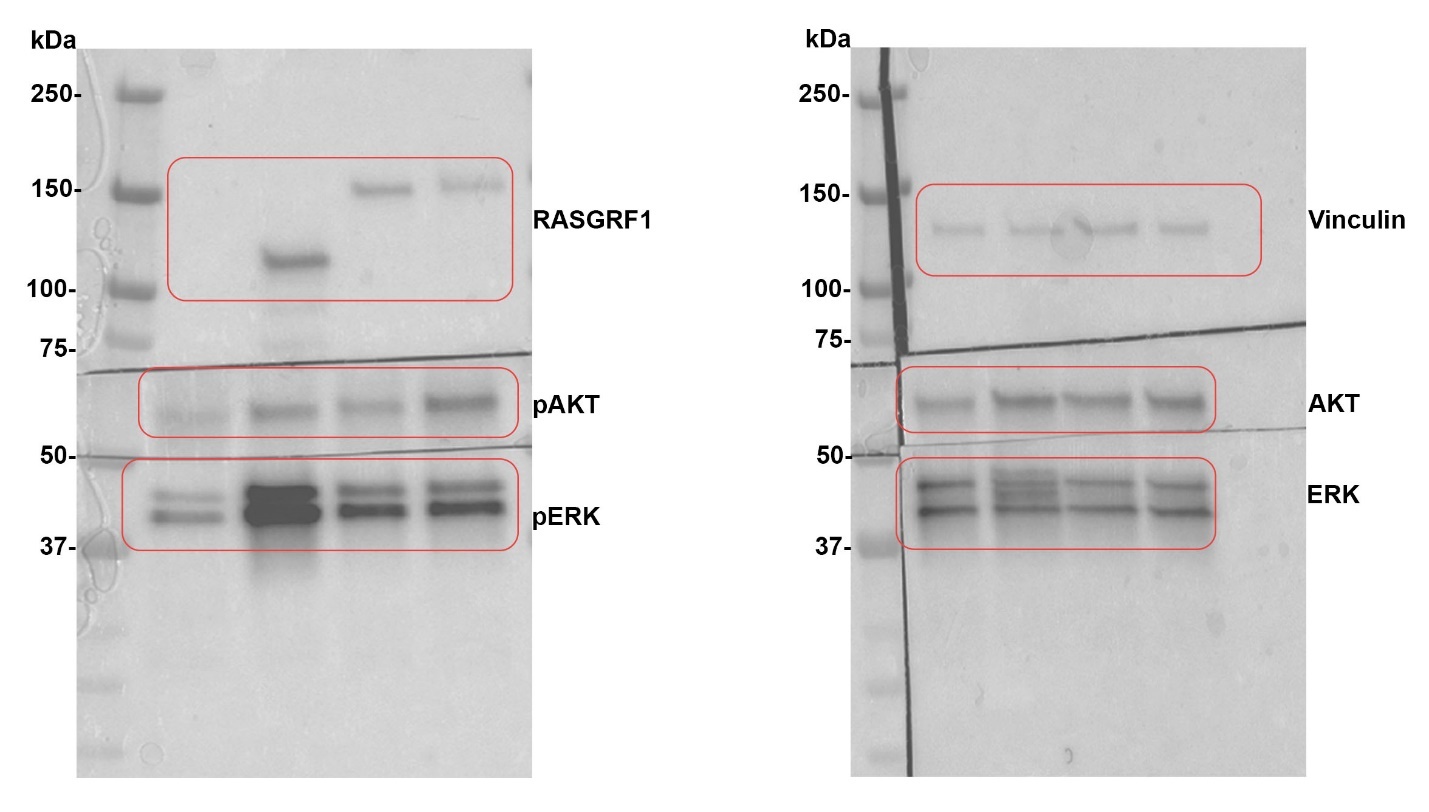
Supplementary Figure 4.** Uncropped images corresponding to Western blot shown in Figure 4e.

**
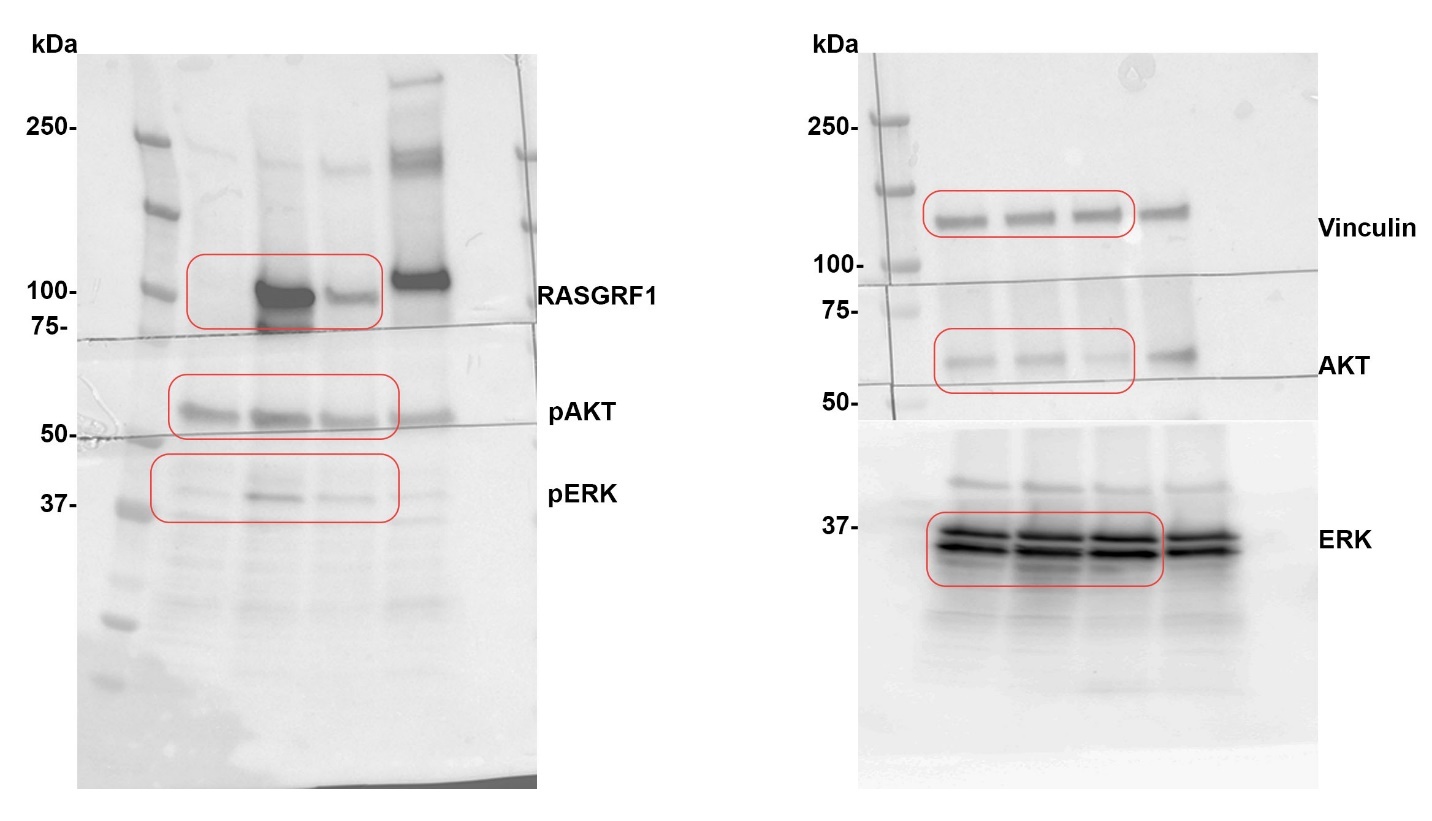
Supplementary Figure 5.** Uncropped images corresponding to Western blot shown in Figure 4h.

**
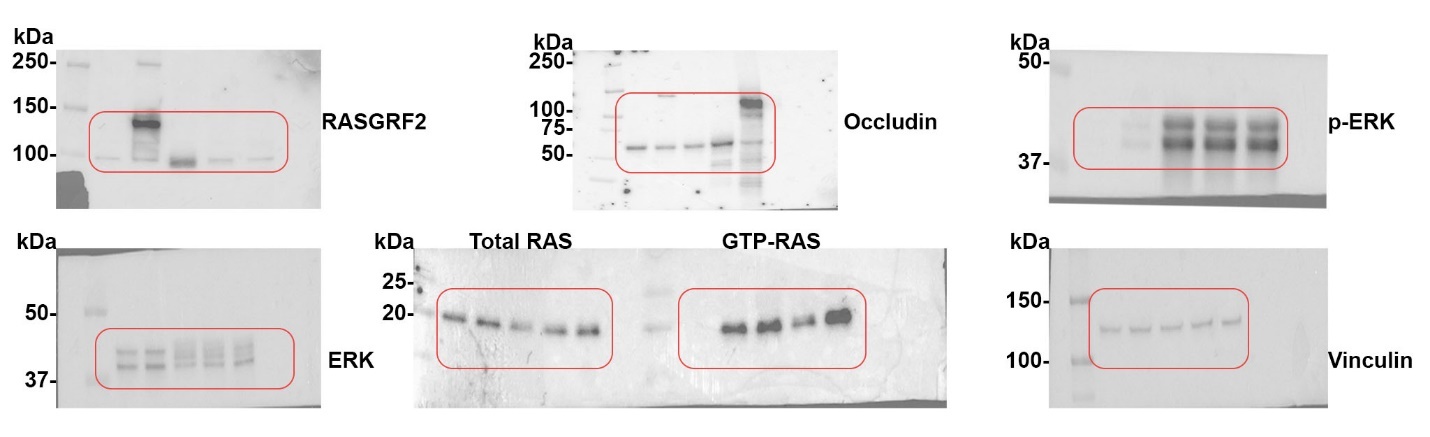
Supplementary Figure 6.** Uncropped images corresponding to Western blot shown in Figure 5b.

**
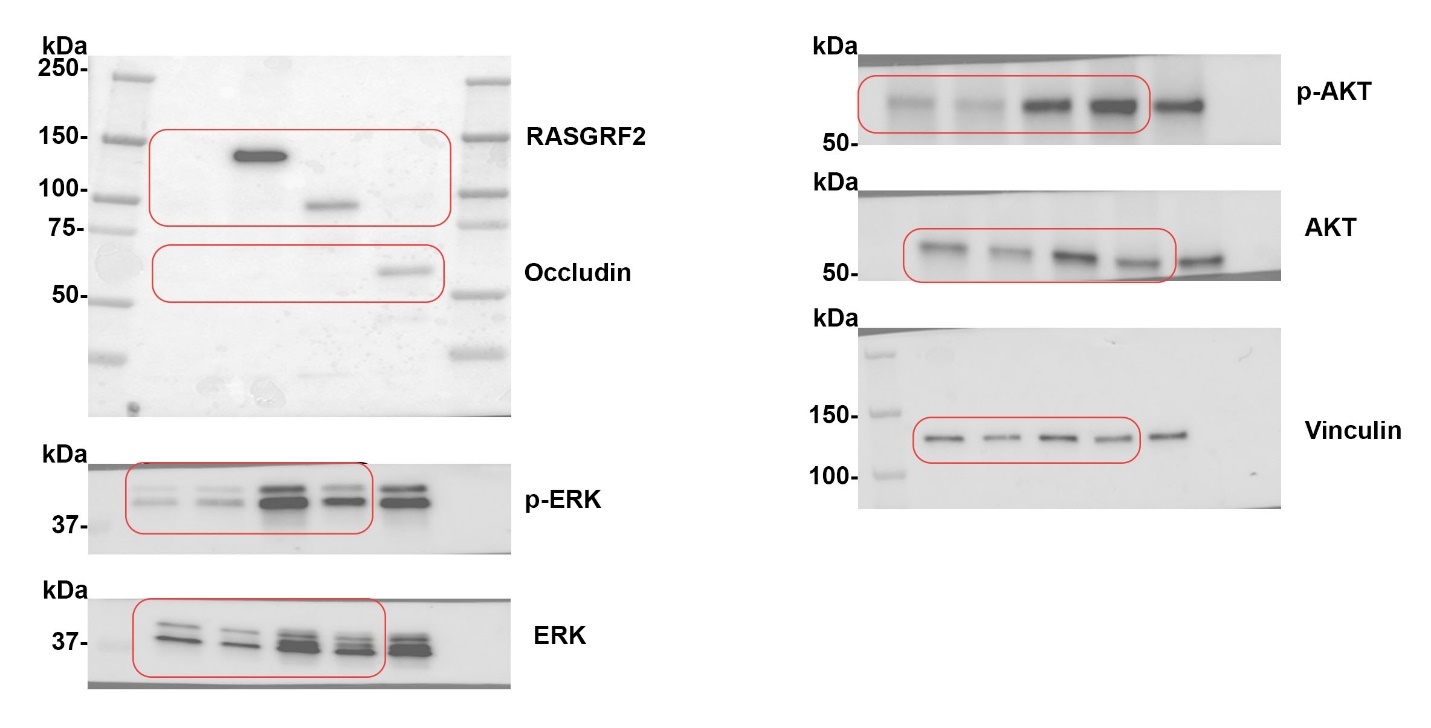
**

**Supplementary Figure 7.** Uncropped images corresponding to Western blot shown in Figure 5e.

**
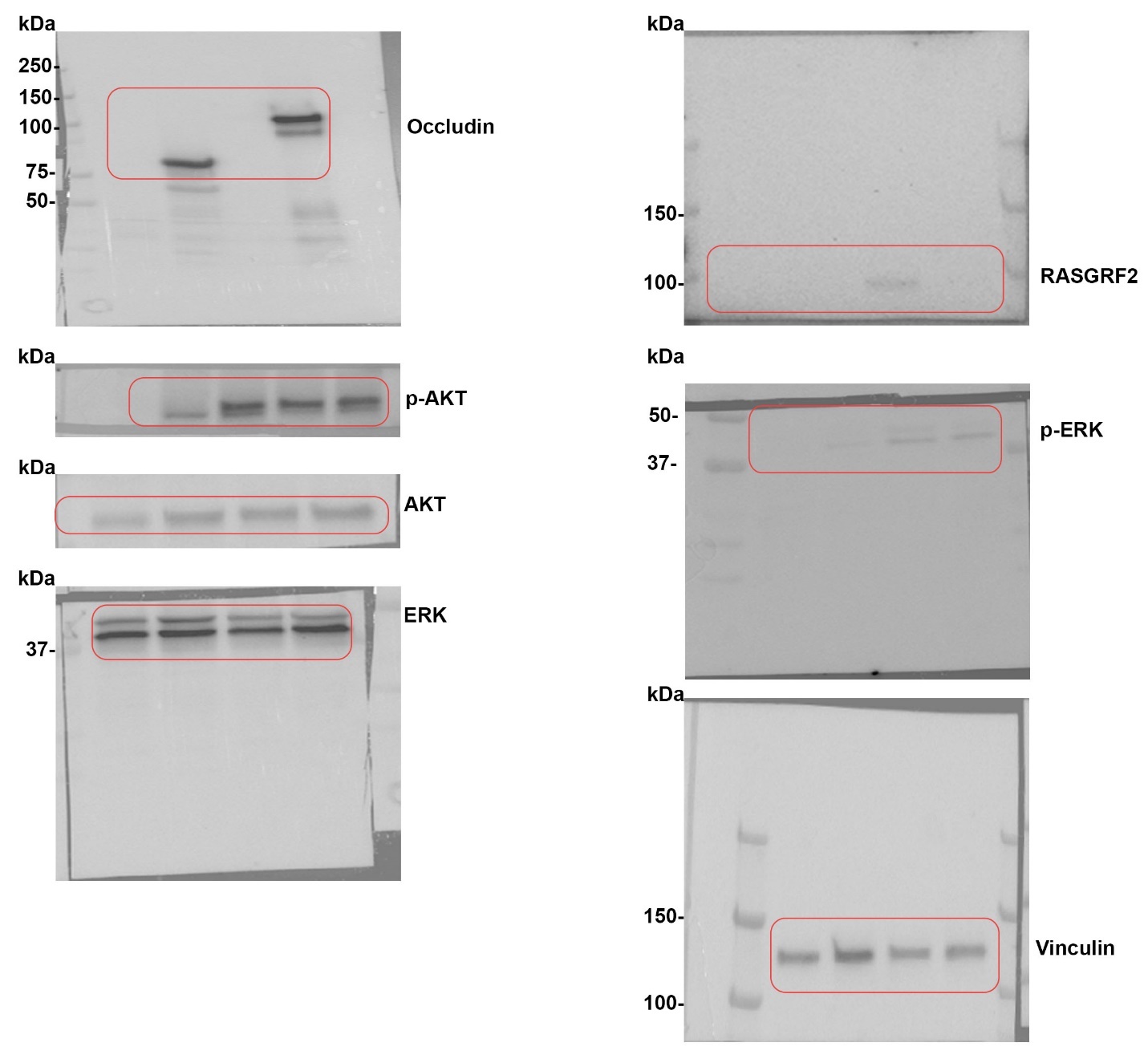
**

**Supplementary Figure 8.** Uncropped images corresponding to Western blot shown in Figure 5g.

**
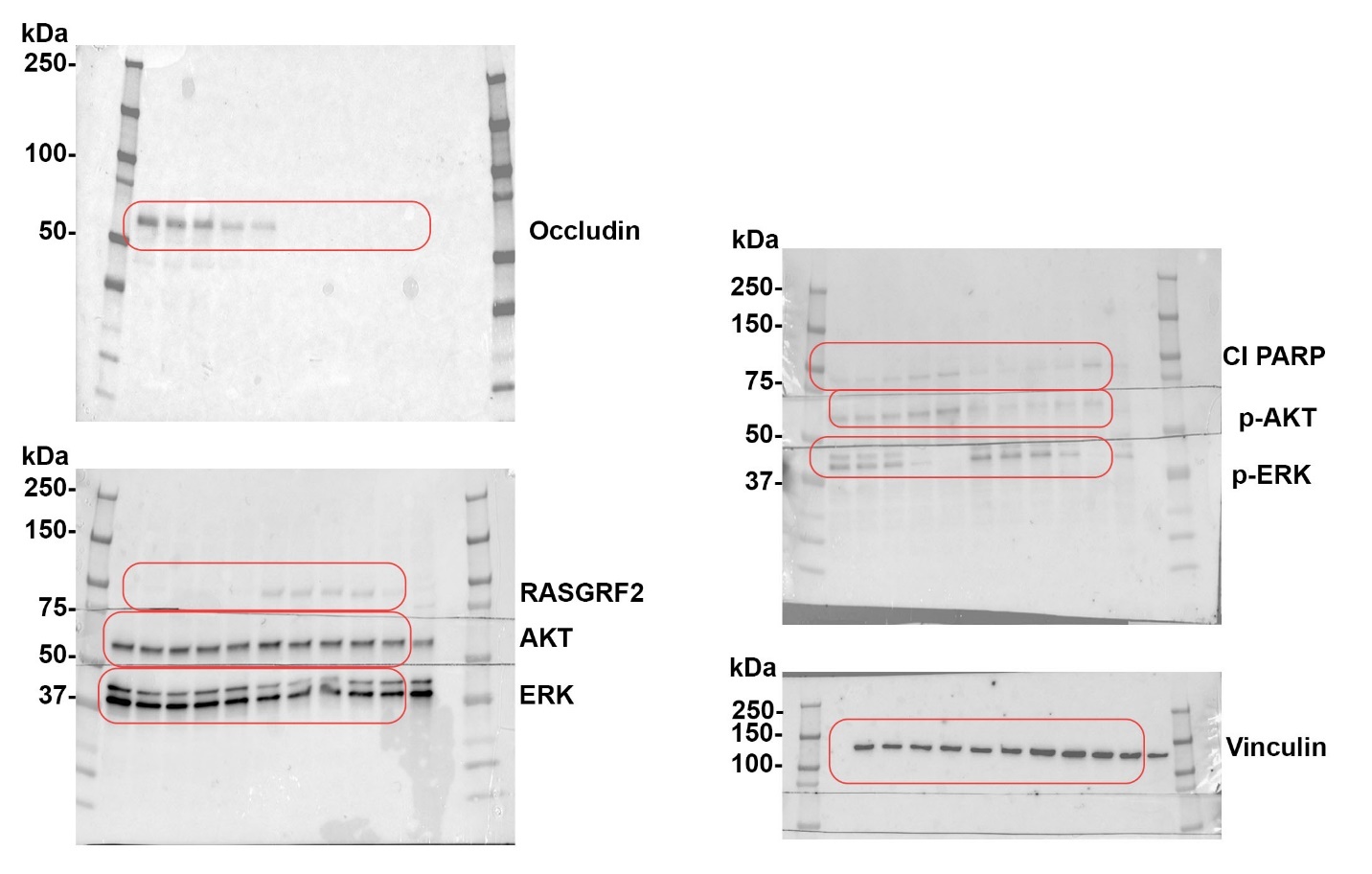
Supplementary Figure 9.** Uncropped images corresponding to Western blot shown in Figure 5i.

**
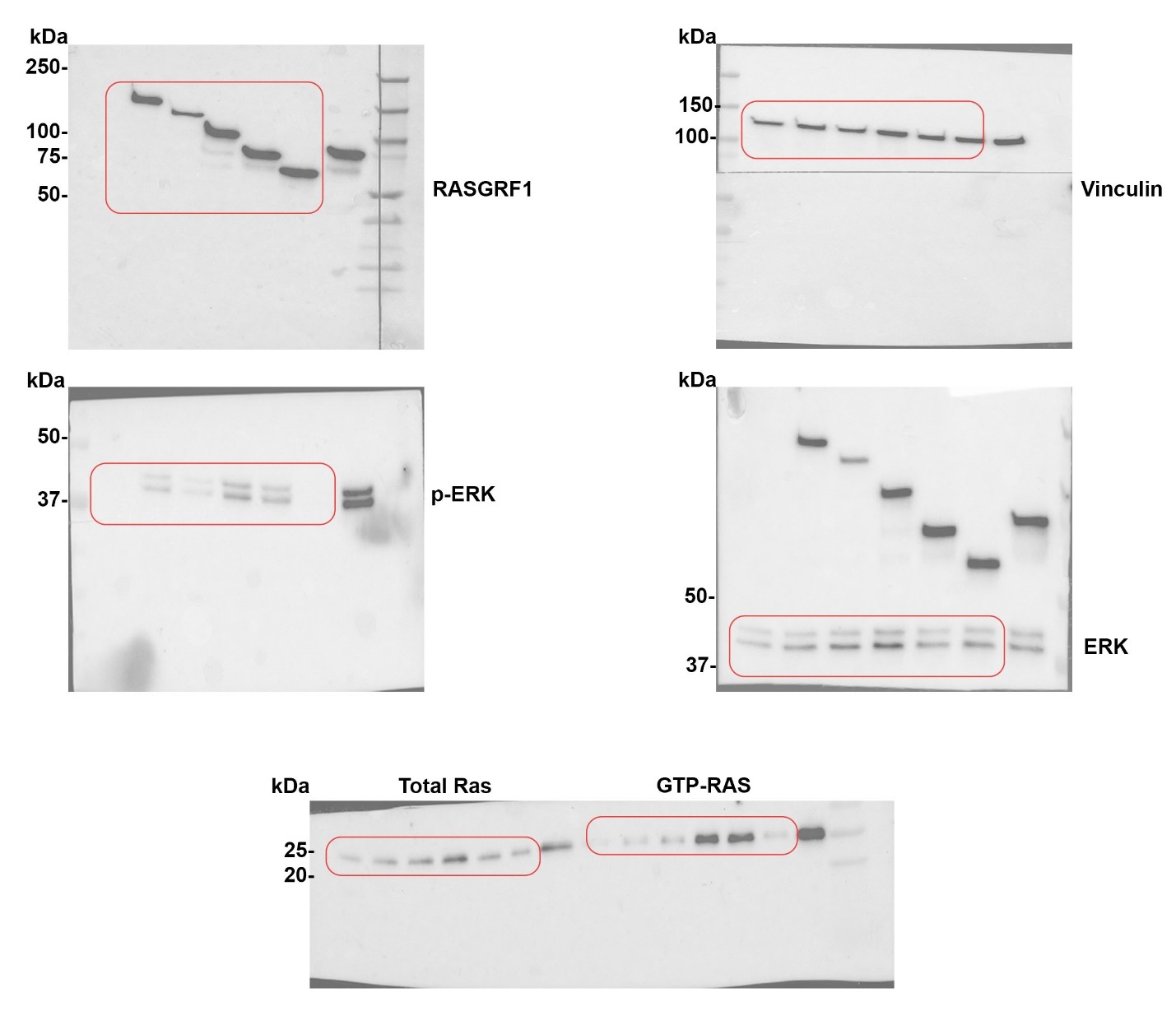
Supplementary Figure 10.** Uncropped images corresponding to Western blot shown in Figure 6b.

**
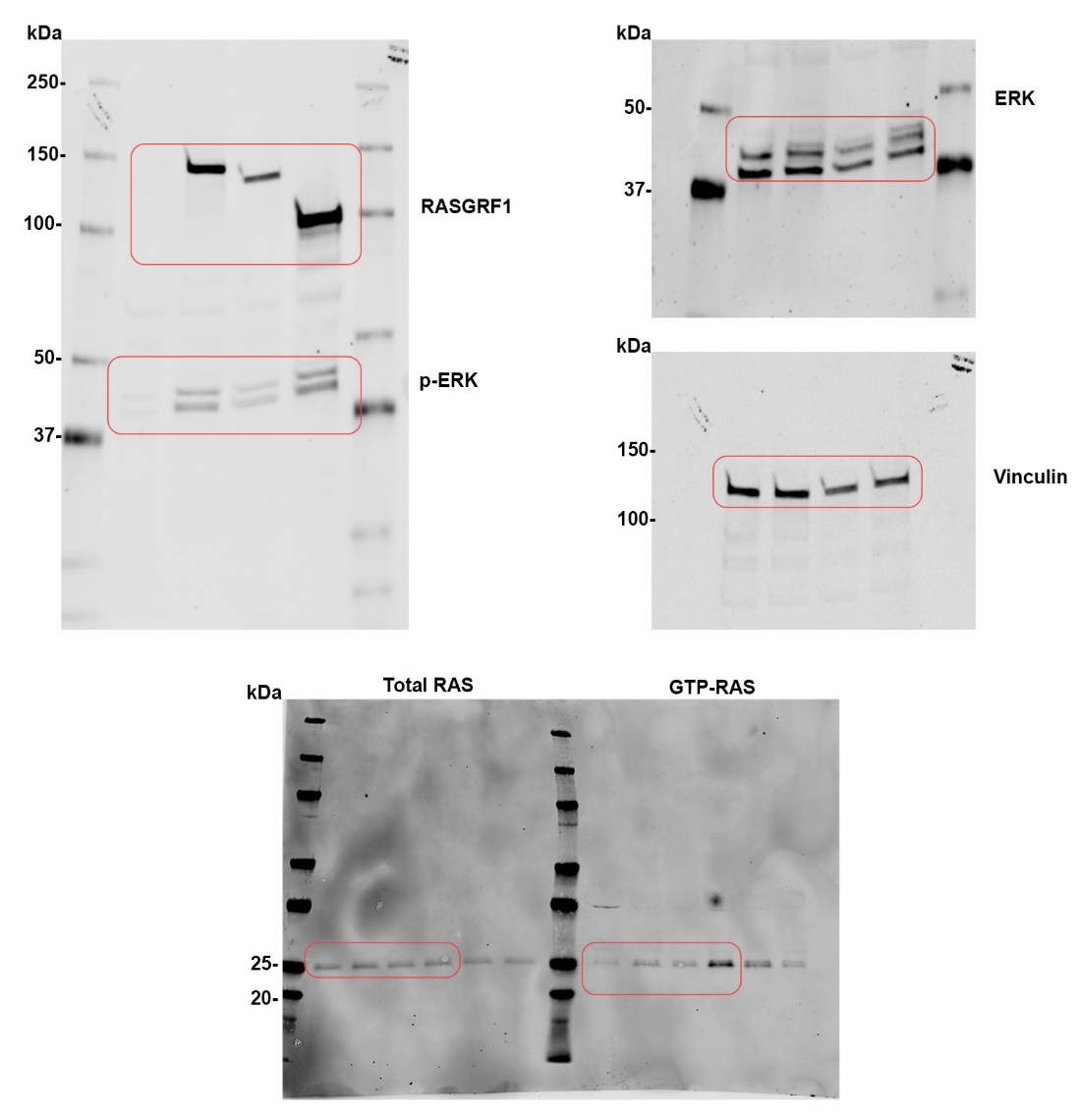
**

**Supplementary Figure 11.** Uncropped images corresponding to Western blot shown in Figure 6c.

**
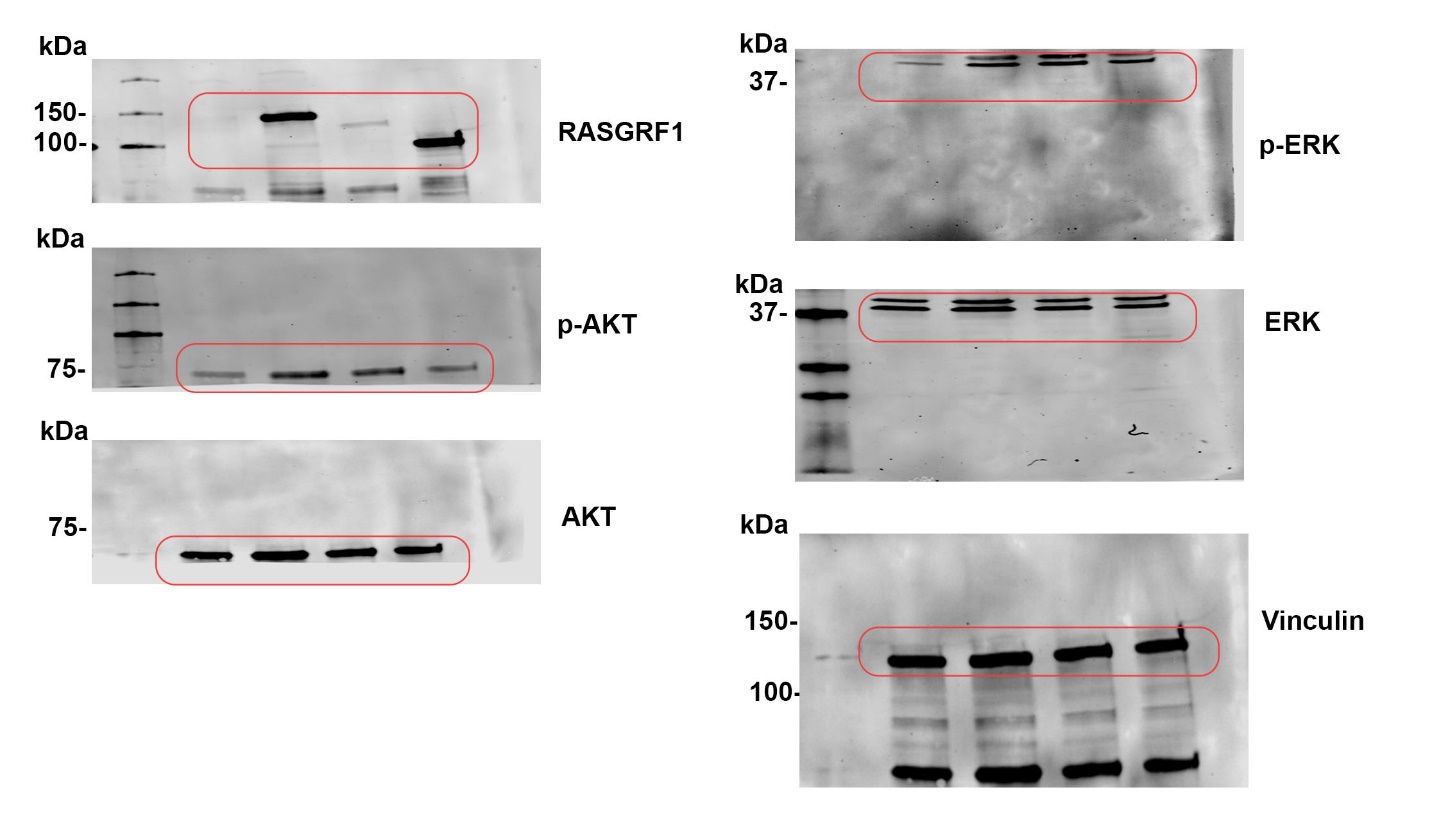
Supplementary Figure 12.** Uncropped images corresponding to Western blot shown in Figure 6f.

**
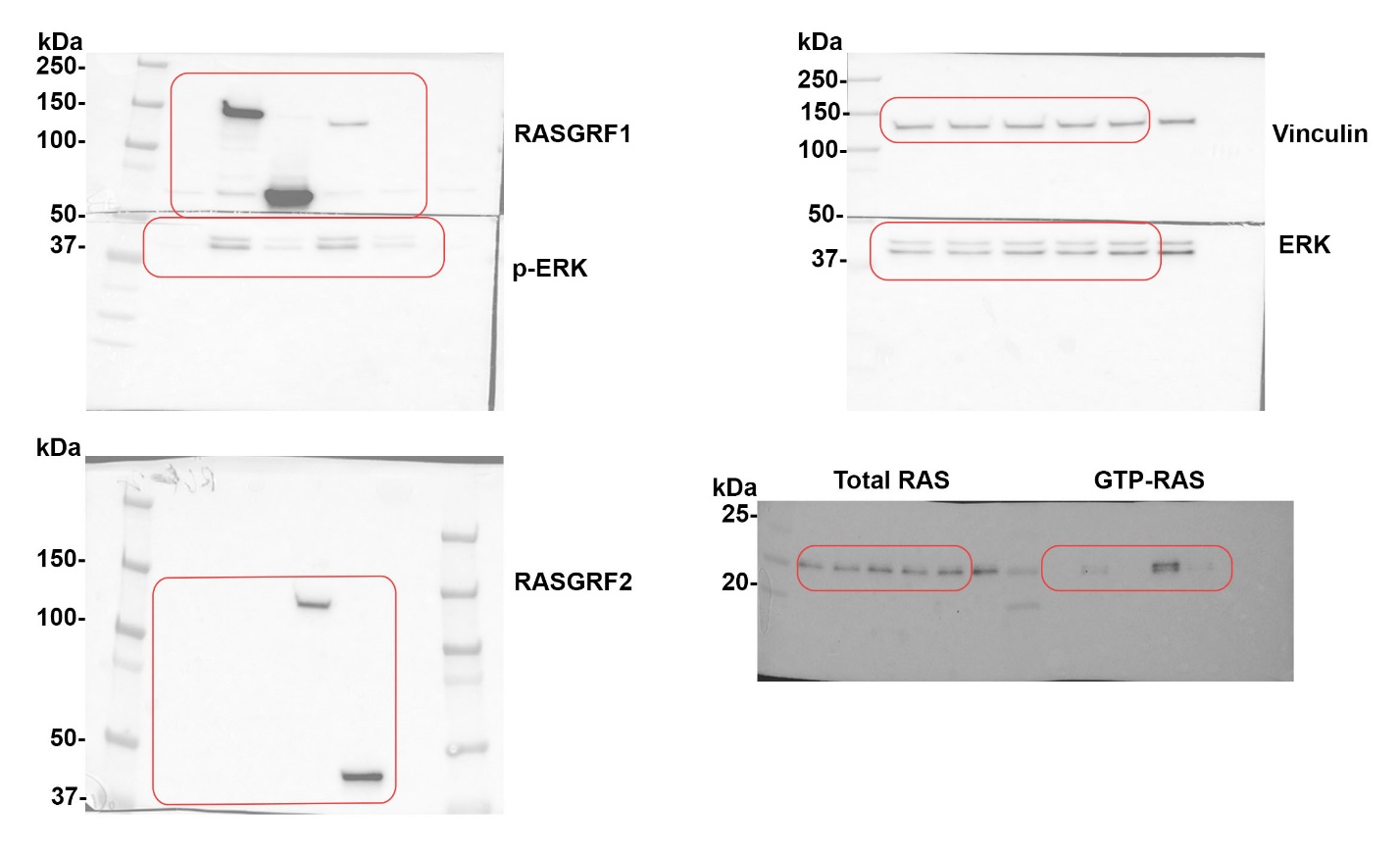
Supplementary Figure 13.** Uncropped images corresponding to Western blot shown in Figure 6g.
